# Super-compression of large electron microscopy time series by deep compressive sensing learning

**DOI:** 10.1016/j.patter.2021.100292

**Published:** 2021-06-24

**Authors:** Siming Zheng, Chunyang Wang, Xin Yuan, Huolin L. Xin

**Affiliations:** 1 Department of Physics and Astronomy, University of California, Irvine, Irvine, CA, USA; 2 Bell Labs, 600 Mountain Avenue, Murray Hill, NJ 07974, USA

**Keywords:** electron microscopy, *in situ*, deep learning, compression, compressive sensing, TEM, big data, direct electron detection, direct detection device

## Abstract

The development of ultrafast detectors for electron microscopy (EM) opens a new door to exploring dynamics of nanomaterials; however, it raises grand challenges for big data processing and storage. Here, we combine deep learning and temporal compressive sensing (TCS) to propose a novel EM big data compression strategy. Specifically, TCS is employed to compress sequential EM images into a single compressed measurement; an end-to-end deep learning network is leveraged to reconstruct the original images. Owing to the significantly improved compression efficiency and built-in denoising capability of the deep learning framework over conventional JPEG compression, compressed videos with a compression ratio of up to 30 can be reconstructed with high fidelity. Using this approach, considerable encoding power, memory, and transmission bandwidth can be saved, allowing it to be deployed to existing detectors. We anticipate the proposed technique will have far-reaching applications in edge computing for EM and other imaging techniques.

## Highlights

• A novel framework, i.e., TCS-DL, has been proposed for big data compressing for EM
• The proposed TCL-DL outperforms JPEG due to the built-in denoising capability
• Considerable power, *in situ* memory, and transmission bandwidth could be saved
• The proposed TCL-DL is a novel and promising way for EM data compressing

## The bigger picture

The rapid development of electron microscopy (EM) opens a new door to exploring physical sciences; however, it raises grand challenges and urgent needs for big data processing. Therefore, it is crucial to compress the EM data. But existing compression methods developed for natural images do not perform well in EM images. In this paper, by combining deep learning and temporal compressive sensing, we propose a novel compression strategy specifically for EM data processing. Owing to the improved compression efficiency and built-in denoising capability of our framework over JPEG compression, compressed videos with compression ratio of 30 can be reconstructed with high fidelity. Therefore, considerable (encoding) power, *in situ* memory, and transmission bandwidth are expected to be saved. In the future, we will strive to increase the compression ratio without reducing the reconstruction quality. And we believe our proposed EM compression method has a wide application for the EM community.

## Graphical abstract

**Figure:**
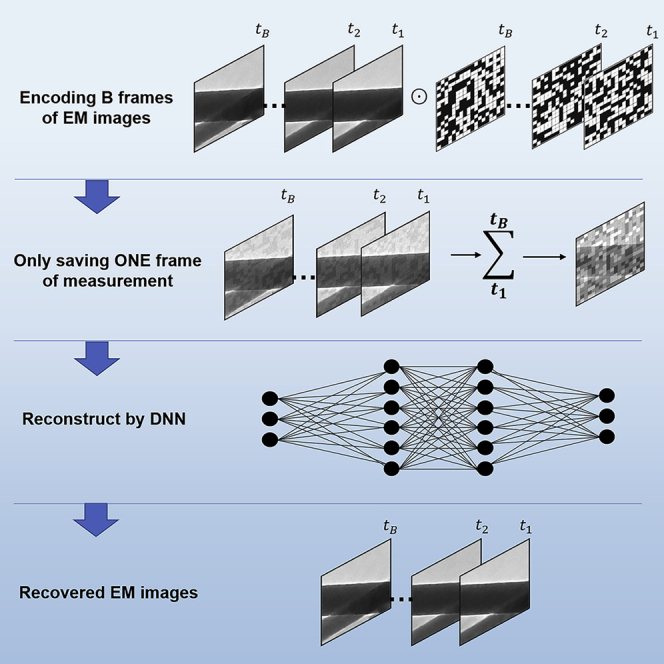

## Teaser

We propose a novel framework, i.e., TCL-DL, for big data compressing for EM. Owing to the significantly improved compression efficiency and built-in denoising capability of our framework over conventional JPEG compression, compressed videos with compression ratio up to 30 can be successfully reconstructed with high fidelity. Therefore, considerable (encoding) power, *in situ* memory, and transmission bandwidth are expected to be saved.

## Introduction

Electron microscopy (EM), as one of the most powerful tools nowadays in probing materials’ structure and chemistry, has extensive applications in biology, physics, chemistry, and materials science owing to its high spatial resolution and chemical sensitivity.^1^ EM provides rich, directly resolved information about the structure and dynamics of phenomena, spanning from the atomic scale to micrometer scale, which are of great fundamental and practical significance to society.^2^ Driven by the recent advances in computer science and electron microscopes, EM techniques, especially *in situ* transmission electron microscopy (TEM),^3^, ^4^, ^5^, ^6^, ^7^, ^8^, ^9^, ^10^ electron tomography,^11^, ^12^, ^13^, ^14^, ^15^, ^16^, ^17^, ^18^, ^19^, ^20^, ^21^, ^22^ four-dimensional scanning transmission electron microscopy (4D-STEM),^23^, ^24^, ^25^ and EM image processing^26^, ^27^ become more and more dependent on big data processing and storage. In particular, due to the deployment of state-of-the-art direct electron detectors, sequential images could be generated with extraordinary frame rates up to thousands of frames per second. This, on the one hand, empowers researchers with the capability to acquire more data with an ultrahigh temporal resolution to discover new phenomena in nature. On the other hand, it poses great challenges to processing, storing, and transmitting large high-resolution EM videos or images. Compressive sensing (CS),^28^, ^29^, ^30^as an efficient signal processing technique, has been widely used in EM for data acquisition and reconstruction. It has been wildly used in capturing high-dimensional data, such as videos^31^, ^32^, ^33^, ^34^ and hyperspectral images.^35^, ^36^, ^37^, ^38^, ^39^, ^40^ As long as the data or signal are compressible or sparse in a certain transform domain, a measurement matrix that is not related to the transform base can be used to project the high-dimensional data obtained by the transformation onto a low-dimensional space, and, by solving an optimization problem, the original data can be reconstructed with high probability from few projections. Extensive attempts^41^, ^42^, ^43^, ^44^, ^45^, ^46^, ^47^ have been made to apply CS to meet the growing demand for efficient EM data acquisition and processing; however, challenges still remain, possibly due to the following drawbacks. On one hand, the iteration-based nature makes the conventional reconstruction algorithms time consuming. On the other hand, some tasks have a higher requirement for hardware, and even the performance could still be unsatisfactory when the compression ratio is higher. Here, by combining CS and deep learning, we propose a novel encoding-decoding strategy to tackle the challenge facing big data EM. Specifically, temporal compressive sensing is first used as an encoder to compress multiple frames into a single-frame measurement with significantly reduced (on the order of 10×) bandwidth and memory requirements for data transmission and storage. An end-to-end deep learning network is then constructed to reconstruct the original image series from the single-frame measurement with extremely high speed. Peak signal-to-noise ratio (PSNR) and structural similarity (SSIM) evaluations show that the temporal compressive sensing-deep learning (TCS-DL) framework exhibits superior performance over the conventional JPEG compression method.

## Results and discussion

**Figure 1:**
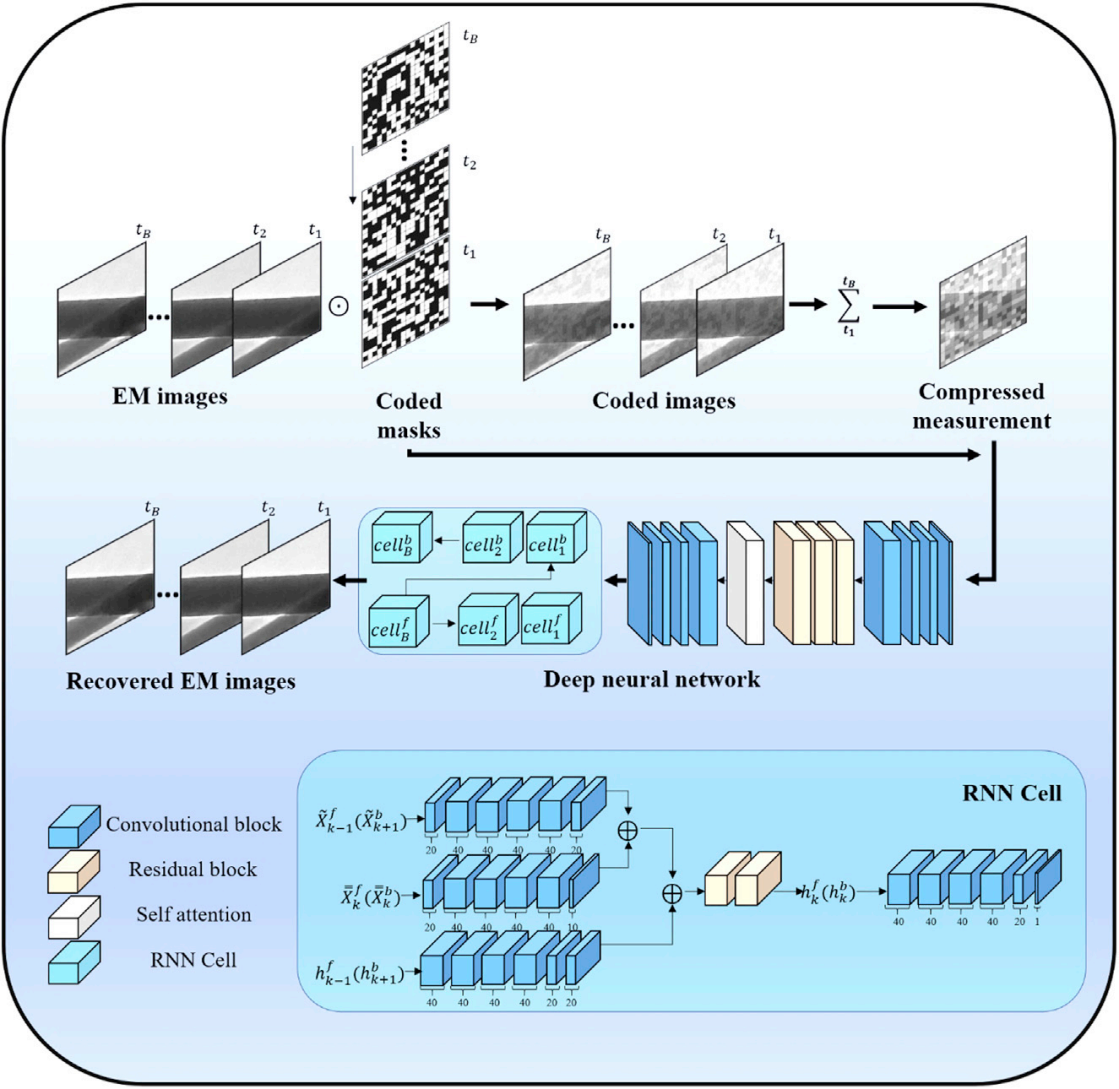
Workflow of the TCS-DL for big data EM The TCS-DL consists of two parts: an encoder compressing *B* frames of EM images using a *B* random binary mask into a single compressed measurement (upper part), and a decoder using a deep neural network to reconstruct the EM images (middle part). The masks used in the encoder and the compressed measurement are first sent into a CNN to generate the first video frame. Then a forward RNN is used to generate the consequent frames. After this, a backward RNN is used to refine the frames in a reverse order to output the final EM images.

The workflow of the proposed TCS-DL approach is summarized (Figure 1). Firstly, for the TCS compression, we consider B continuous video frames (*B* represents the number of EM images) captured at time slots $t_{1},\;t_{2},\ldots ,t_{B}$. For each frame, we superimpose a random binary mask (coded masks) composed of 0 and 1 with the same probability. The coded images are then summed to form a single-frame measurement,^31^^,^^48^ denoted by compressed measurement in the top right of Figure 1. Until now, the encoder part of the TCS-DL is finished with B EM images being compressed to a single-frame compressed measurement. Since the B -coded masks can be pre-determined and stored, the data size is reduced by B times. A multi-layer deep learning network is then constructed and employed as a decoder to reconstruct the original multi-frame images from the single-frame measurement. The deep neural network (DNN) is composed of a convolutional neural network (CNN) plus two recurrent neural networks (RNNs) (see details in the methods). During our experiments, the summing process in the encoder does not squeeze the dynamic range of the original image. It is purely a mathematical summation process. As we mentioned before about CS, the compressed process can be expressed as y=Φx, where y is the measurement, Φ is the compression matrix, and *x* are the original frames. Reconstructing *x* from *y* is an ill-conditioned problem. In this work, the optimization problem is solved by a DNN. It combines the CNN and RNN, which takes into account the correlation between adjacent frames; the learned correlation will help to reconstruct the result by maintaining the information included in the original images.

**Figure 2:**
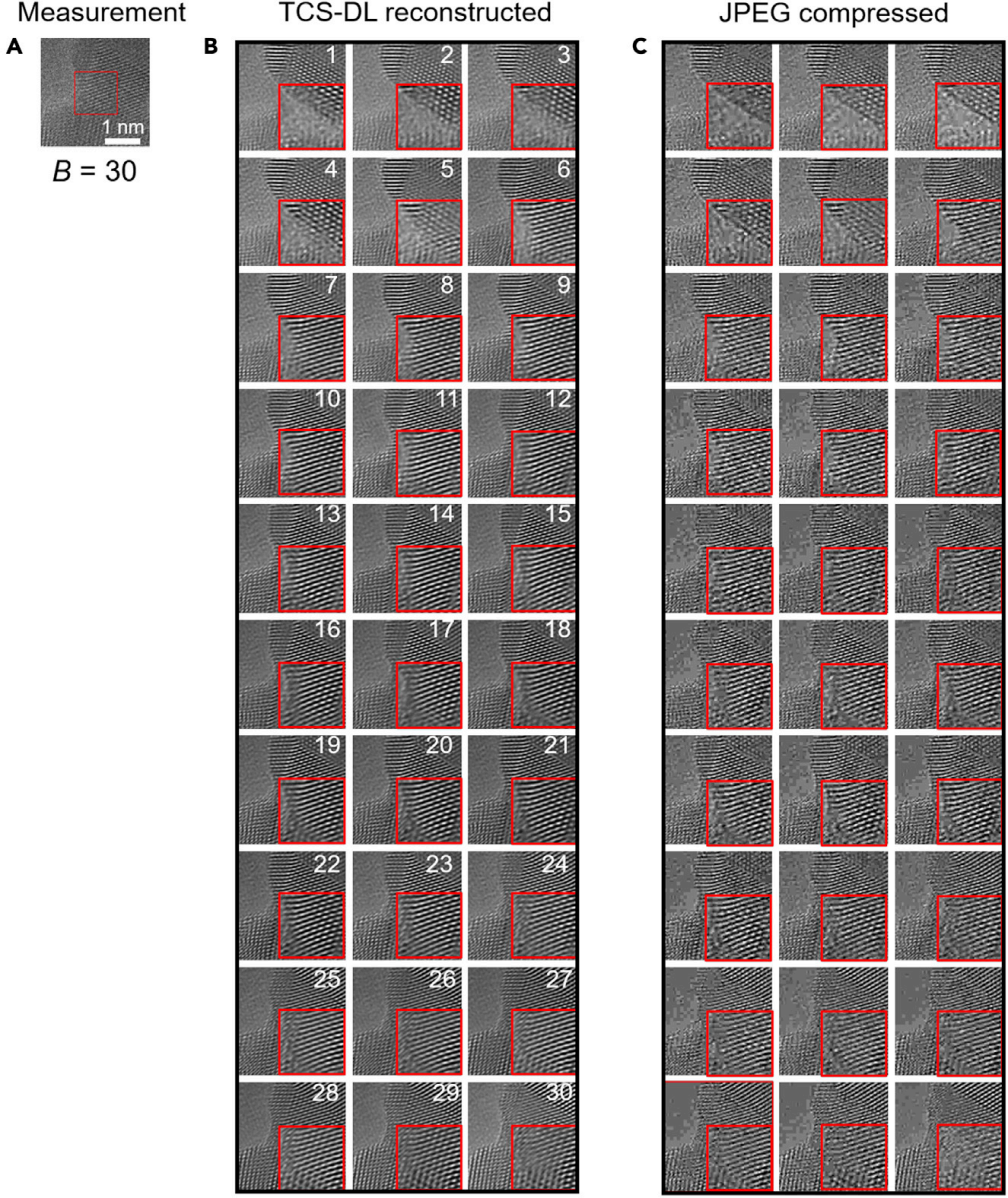
Performance of TCS-DL framework on *in situ* atomic-resolution EM images (A) The single-frame compressed measurement obtained from 30 sequential images using TCS-DL. (B) Reconstructed sequential images obtained from the compressed measurement in (A) using TCS-DL. The average PSNR is 25.91 dB compared with the denoised raw EM images. (C) JPEG-compressed images with the total image size equal to the compressed measurement in TCS-DL. The average PSNR is 23.98 dB. Insets show magnified images of local regions (raw images in Figure S6).

The performance of TCS-DL is tested on sequential atomic-resolution images of Au nanocrystals with complicated structures. These images, captured at full resolution (4k × 4k), show the *in-situ-*sintering process of Au nanocrystals during high-resolution TEM imaging (Figure 2). The raw images are processed using the TCS-DL framework with a compression rate *B* = 30, that is, 30 sequential EM images are compressed into a single frame measurement (Figure 2A) and then reconstructed (Figure 2B). As a comparison, JPEG compression is also performed on the raw images (Figure 2C). Note that, to keep the memory consumption to store the images the same as that using TCS-DL, the size of the JPEG-compressed image is kept as 1/*B* of the original image size. The results show that obvious artifacts and noises are observed in the JPEG-compressed images. In sharp contrast, the reconstructed EM images using TCS-DL show a clean background, fine details, and sharp edges. To quantitatively evaluate the performances of TCS-DL and JPEG, we calculate the PSNR and SSIM of both JPEG-compressed images and TCS-DL reconstructed images compared with denoised raw images. Note that these raw images are usually noisy and a denoising algorithm^49^^,^^50^ can be applied. In this paper, we use BM3D for denoising. However, this will incurs power and computation costs. Therefore, in this work, both JPEG and TCS-DL are performed directly on the raw images. The average PSNR for JPEG compression and TCS-DL is 23.98 and 25.91 dB, respectively. The PSNR improvement of 1.93 dB indicates an evidently improved performance of TCS-DL over JPEG, which is in good agreement with the enhanced image quality using TCS-DL (Figure 2B) compared with JPEG compression (Figure 2C).

**Figure 3:**
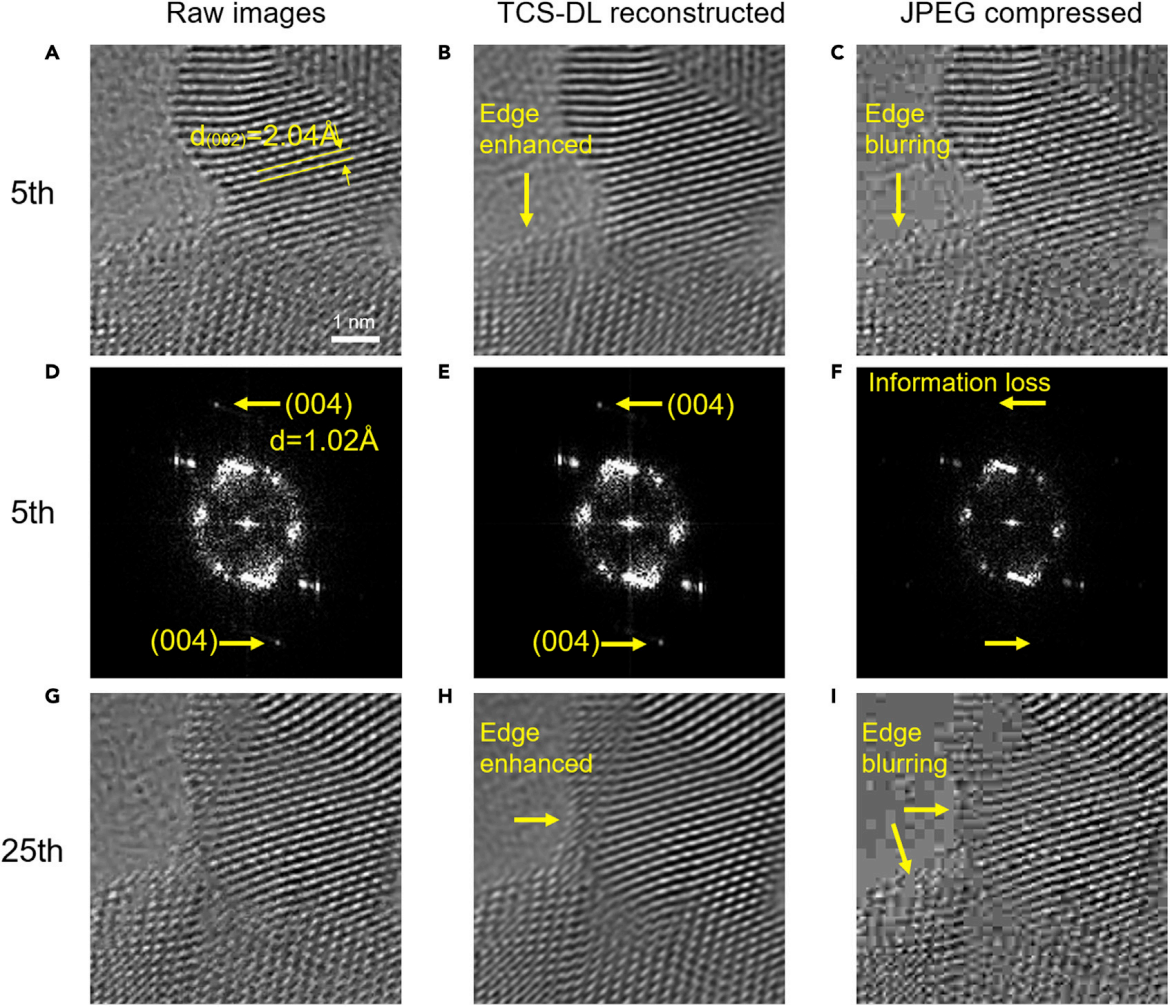
Comparison of selected atomic-resolution images obtained using TCS-DL and JPEG compression in Figure 2 (A–C) 5^th^ raw images, reconstructed images using TCS-DL and JPEG compression. (D–F) Fast Fourier transforms of the images in (A–C). Bragg spots corresponding to the (004) planes are indicated by the arrows in (D and E). Information loss is identified in the JPEG-compressed image (F) as the Bragg spots corresponding to (004) planes are not resolved. (G–I) 25^th^ raw images, reconstructed images using TCS-DL and JPEG compression. Compared to the raw image (G), more surface details are observed in the TCS-DL-processed image (H) due to the edge enhancement. In contrast, deteriorated image quality such as edge blurring is identified in the JPEG-compressed image (I).

A more detailed comparison is made on representative selected frames as shown in Figure 3. The reconstructed images show enhanced surface atomic details (indicated by the arrows in Figures 3B and 3H) compared with that in the raw image (Figure 3A). However, in sharp contrast, the JPEG-compressed image shows severely deteriorated atomic details, especially at the crystal surface, e.g., the edge blurring as indicated by the arrows (Figures 3C and 3I). Fast Fourier transform (FFT) is further performed on the raw image as well as that processed using TCS-DL and JPEG compression (Figures 3D and 3F). The result shows that the high-frequency information (e.g., the (004) plane of the Au nanocrystal) in the raw image is well retained in the reconstructed image using TCS-DL. In contrast, the FFT of the JPEG-compressed image shows deteriorated resolution (indicated by the unresolved (004) plane of the Au nanocrystal) compared with the raw image and reconstructed image using the TCS-DL framework. The above results reveal that, distinct from the conventional JPEG, which introduces information loss during compression, the TCS-DL framework is capable of recovering the detailed information in the raw data. In addition, the built-in denoising capability of TCS-DL could further slightly improve the quality of raw images.

**Figure 4:**
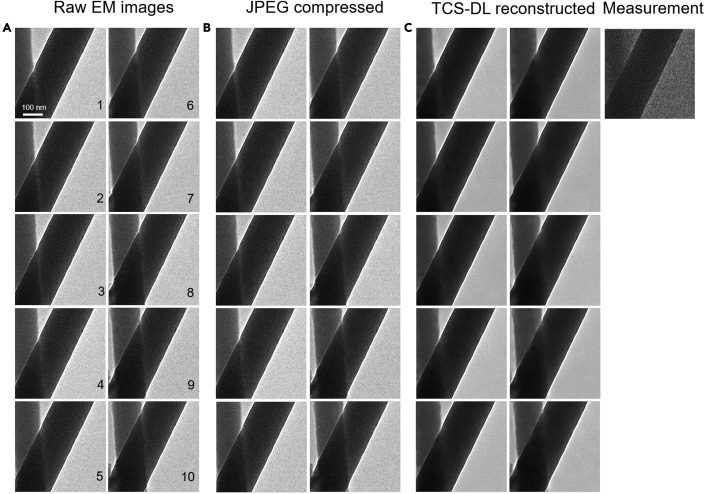
Performance of the TCS-DL framework on carbon fibers with relatively simple morphology (*B* = 10) (A) Raw images of the carbon fibers during *in situ* deformation. (B) JPEG-compressed images with the total image size equal to the compressed measurement in TCS-DL. The average PSNR is 32.38 dB compared with the denoised raw images. (C) Reconstructed images using the TCS-DL framework. The average PSNR is 35.41 dB. Note that the JPEG-compressed images preserve the noise in the raw images (additional results in Figures S3–S5).

Aside from atomic-resolution images of ultrasmall nano-objects, we further tested the performance of the TCS-DL framework on objects with a much larger feature size in a large field of view. Figure 4 shows sequential EM images of two carbon fibers during *in situ* deformation. The raw images are processed using TCS-DL and JPEG compression, respectively. The TCS-DL reconstructed images show a clean background as well as a sharp edge, which thereby enables accurate tracking of the motions of the fibers (Figure 4C). In contrast, the JPEG-compressed images inherit the noises in the raw images, similar to that in atomic-resolution images as shown in Figures 2 and 3. It is worth noting that, due to the relatively simple morphology of the carbon fibers compared with Au nanocrystals, the PSNR of both JPEG compression and TCS-DL framework are evidently higher than that of the atomic-resolution images, as shown in Figures 2 and 3. Yet, the TCS-DL framework still outperforms JPEG compression with a considerable PSNR improvement of approximately 3 dB, from 32.38 dB (JPEG compression) to 35.41 dB (TCS-DL). Similar performance improvement is also obtained for EM images reconstructed from TCS-DL at *B* = 20 (Figure S1). Results of different compression ratios are shown in Video S1. We also present the results of different video compression algorithms in Videos S2 and S3.

Video S1. Performance under different compression ratio of TCS-DL framework on *in situ* atomic-resolution EM images

Video S2. Performance of avi compression algorithm on *in situ* atomic-resolution EM images

Video S3. Performance of mp4 compression algorithm on *in situ* atomic-resolution EM images

**Figure 5:**
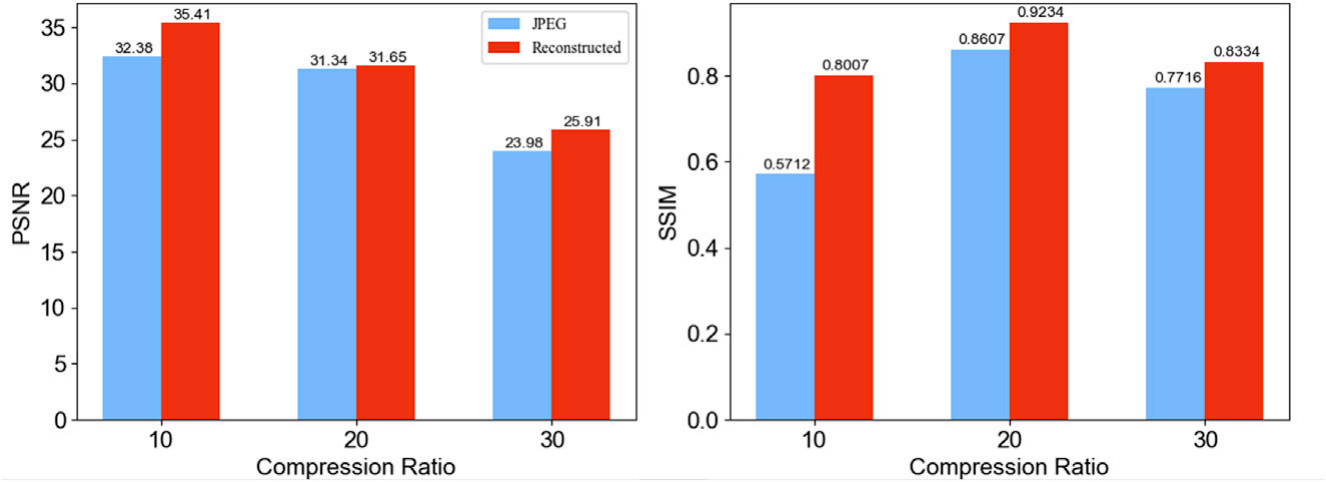
Average PSNR (in dB), SSIM of TCS-DL and JPEG at different compression ratios Denoised raw EM images are used as a baseline for calculation of the PSNR and SSIM. The results are based on the data in Figures 3, 4, and S2.

PSNR and SSIM are calculated and summarized to quantitatively compare the performance of the TCS-DL framework and JPEG compression. As shown in Figure 5, we conduct both TCS-DL and JPEG compression at different compression ratios from *B* = 10 to *B* = 30. PSNR, which is a ratio between the maximum possible power of a signal and the power of corrupting noise that affects the fidelity of its representation, is mainly used to evaluate the performance of reconstructed EM images using different compression methods. SSIM is used for measuring the similarity between the reconstructed EM images and the corresponding ground truth. Clearly, TCS-DL has higher PSNR and SSIM, which means that it can get better performance, especially when the raw images are noisier (Figures 2 and 4) and the compression ratio (Figure 2) is high, because our framework can reduce the background noise while JPEG produces obvious artifacts when the compression ratio is high. What needs to be mentioned is that the reconstruction process in this work utilizes both CNN and RNN structures, where RNN is mainly used to learn the correlation between adjacent frames. If the frames are obviously incoherent, the reconstruction result would be vulnerable. In most instances, the frames in the *in situ* TEM movie are coherent. We can utilize our compression method under the condition just mentioned. But at the edge, when the content in the receptive field changes suddenly, we do not use this method to compress the incoherent frames. In future work, we can automatically detect the sudden change and choose the corresponding compression method. If the receptive field does not change frequently, which means that the coherent frame number is sufficiently greater than the compression ratio *B*, TCS-DL can achieve a generally impressive result.

**Figure 6:**
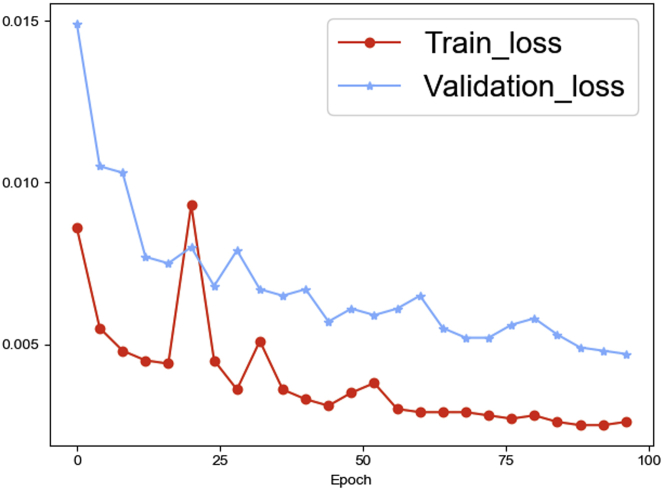
The training loss and validation loss during the learning process with *B* = 10 The validation loss gradually converges as the training process progresses.

The training loss and validation loss during the learning process with *B = 10* are shown in Figure 6. As we can see, the validation loss gradually converges as the training process progresses. Because we have sufficient data and we stop the training at the appropriate epoch, the validation loss curve does not show a V-shaped trend,which means it is not overfitted.

### Conclusion

In conclusion, by combining deep learning and temporal compressive sensing, a novel encoding-decoding framework, i.e., TCS-DL, has been proposed for big data compression/decompression for EM. Owing to the significantly improved compression efficiency and built-in denoising capability of the TCS-DL framework over the conventional JPEG compression method, compressed EM time series with a high compression ratio can be successfully reconstructed with high fidelity by the TCS-DL framework from a single measurement. Our work, by offering a universally applicable super-compression strategy for EM data compression, opens the possibility for EM big data storage, processing, and transmission at the expense of significantly reduced power, *in situ* memory, and transmission bandwidth.

## Experimental procedures

### Resource availability

#### Lead contact

Further information and requests for resources and reagents should be directed to and will be fulfilled by the lead contact, Huolin L. Xin (huolinx@uci.edu).

#### Materials availability

This study did not generate new unique reagents.

#### Data and code availability

The original data within this paperis available from our website http://temimagenet.ps.uci.edu/. The code used in this work is available at https://github.com/xinhuolin/TCS-DL.

### Methods

We first derive the mathematical model of TCS-DL and then present the details of the DNN for reconstruction.

Let $X_{1},\ldots ,\;X_{B}$ denote the B frames of the EM images being coded with masks $C_{1},\ldots ,\;C_{B}$. Each frame is of spatial size $N_{x}\times N_{y}$ pixels. From Figure 1, the coded measurement can be expressed as

(Equation 1) $$Y=\sum_{b=1}^{B}X_{b}⊙C_{b}.$$

Here, ⊙ denotes the element-wise product. Note that since the masks are composed of 0 and 1, there is no multiplication in Equation 1. We only need to index the “1” elements in the corresponding frames and sum across the B frames to get the compressed measurement Y. Therefore, our proposed encoder is ultra-simple. Indeed, only Equation 1 will finish the encoder and the complexity is of O(1).

The decoder aims to estimate the EM images, which can be denoised based on the previous analysis, from Y, given masks. As shown in Figure 1, we use a CNN and two RNNs to perform the reconstruction.^51^^,^^52^

The more information RNN takes as input, the better results we get. Therefore, the first frame for RNN with as much visual information as possible is required. To this purpose, we utilize a CNN with 12 layers to reconstruct the first input frame. We introduce normalized measurement $\bar{Y}$

(Equation 2) $$\bar{Y}=Y⊘\left(\sum_{k=1}^{B}C_{K}\right)$$

as input component of CNN. Where ⊘ denotes the matrix division. We concatenate $\bar{Y},\bar{\;Y}⊙C_{1},\;\bar{Y}⊙C_{2},\ldots \bar{Y}⊙C_{B}$ along the third dimension. The concatenation result is then fed into the CNN which consists of two four-layer CNNs, one three-layer residual block,^53^ and one self-attention block.^54^ Then, the first frame ${\tilde{X}}_{1}^{f}$ for RNN is generated. It is worth noting that $\bar{Y}⊙C_{K}$ is to approximate the real coded frames.

After getting ${\tilde{X}}_{1}^{f}$, we introduce two RNNs with different directions to reconstruct the frames. As shown in Figure 1, the input of each RNN cell consists of three different parts. The first part is got from the former RNN cell's output ${\tilde{X}}_{k-1}^{f}\left({\tilde{X}}_{k+1}^{b}\right)$ except the first ${\tilde{X}}_{1}^{f}$, which is obtained from the CNN. The second part ${\bar{\bar{X}}}_{k}^{f}\left({\bar{\bar{X}}}_{k}^{b}\right)$ is acquired by

(Equation 3) $${\bar{\bar{X}}}_{k}^{f}=\bar{Y}⊕\left(Y-\sum_{t=1}^{k-1}C_{k}⊙{\tilde{X}}_{t}^{f}-\sum_{t=k+1}^{b}C_{t}⊙\bar{Y}\right),$$

where ⊕ denotes concatenation along the third dimension which has the same meaning in Figure 1. The second item in the right part of Equation 3 could be considered to be an approximation of $C_{k}⊙X_{k}$ to some extent. The third part $h_{k-1}^{f}\left(h_{k+1}^{b}\right)$ is hidden units obtained by former RNN cell except the first one $h_{1}^{f}$ initialized by zero. The three input parts are separately fed into three sub-CNNs. After the process of concatenation, there are two residual blocks, which outputs the new hidden unit fed to another sub-CNN. Then, each cell outputs the reconstructed frame. Noting that forward and backward RNNs have the same structure, but they do not share the parameters. Besides, the second input part of the backward RNN cell is different from the forward RNN cell

(Equation 4) $${\bar{\bar{X}}}_{k}^{b}=\bar{Y}⊕\left(Y-\sum_{t=B,t\neq k}^{1}C_{k}⊙{\tilde{X}}_{t}^{f}\right),$$

which uses reconstruction ${\tilde{X}}_{k}^{f}$ from forward RNN instead of $\bar{Y}$. Another difference is that the initial hidden unit for backward RNN is not set to zero but $h_{B}^{f}$ which contains more information.

## Supplemental information

Document S1. Supplemental experimental procedures and Figures S1–S6

Document S2. Article plus supplemental information
