# Supplementary material for: Super-compression of large electron microscopy time series by deep compressive sensing learning
Source: Patterns (N Y). 2021 Jun 24;2(7):100292. doi: 10.1016/j.patter.2021.100292 (PMC8276025; doi:10.1016/j.patter.2021.100292)
Supplement: Document S1. Supplemental experimental procedures and Figures S1–S6 [file mmc1.pdf]

Patterns, Volume *2*

Supplemental information

Super-compression of large electron microscopy time series by deep compressive sensing learning

Siming Zheng, Chunyang Wang, Xin Yuan, and Huolin L. Xin

SUPPLEMENTAL EXPERIMENTAL PROCEDURES

Training Details

Our reconstruction network is trained on the data captured by ourself with the pixel resolution of 2048*2048 and published video data on the internet. We cropped patches of size 256x*256 with 3 different compression ratios (CR). The respective number of patches for each network with B = 10, 20, and 30 is 3000, 3500 and 4000. We use PyTorch for implementation and Adam as the optimizer. The total number of training epochs is respectively set to 100, 150, and 250 with a learning rate of $5*10^{-4}$ which decays 10% every 10 epochs before epoch 100 and decays 5% every 20 epochs after epoch 100. Training the network with B=10 took approximately 5 days which is run on a Nvidia GTX 1080 Ti GPU with 11GB RAM. The networks with B = 20, 30 are all run on the NVIDIA RTX 8000 GPU with 48GB RAM which take respectively approximately one and three weeks.

Decoding Time

As shown in Fig. S1, TCS-DL can reconstruct EM images efficiently. We also compare with another deep learning reconstruction method U-net only with B = 10. It is a little bit faster than TCS-DL, but it does not take adjacent frames' correlation into consideration and the model is fixed so that it can not get as satisfactory reconstruction results as TCS-DL when the compression ratio increases. As we can see, the decoding time increases as the compression ratio gets higher for the reason that TCS-DL's parameters increase as the compression ratio gets higher. In spite of the decoding time is got at the resolution of 256*256, TCS-DL can be paralleled on multiple GPUs which only has negligible latency, we can reconstruct EM images with larger resolution in approximately the same time. Besides we could apply TensorRT to speed up TCS-DL by inference optimization in practice.

Data Availability

The datasets and source codes to reproduce the results are available from the corresponding author on reasonable request.

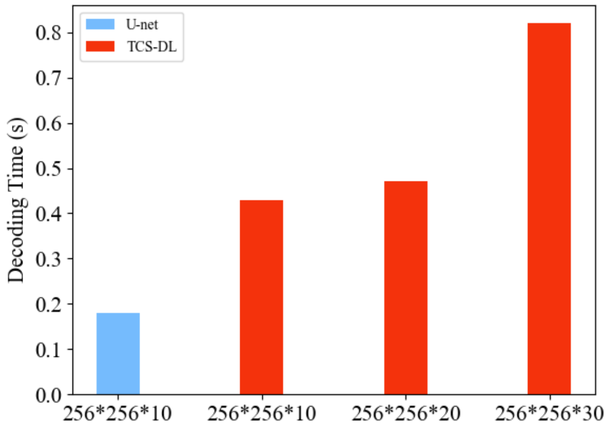

Figure S1. Decoding time of TCS-DL with *B* = 10, 20, and 30 and U-net with *B* = 10.

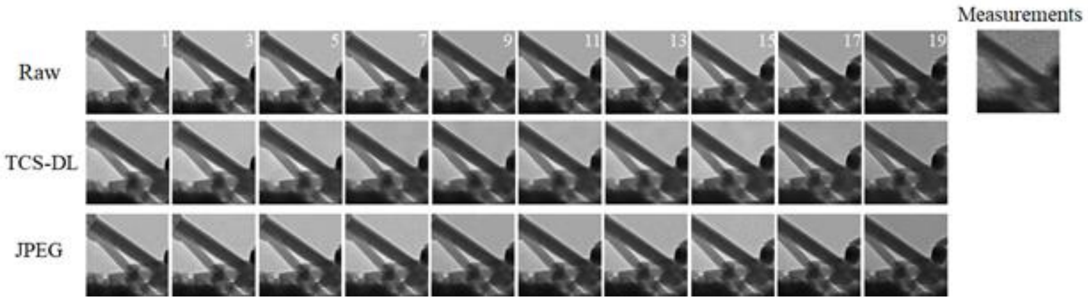

Figure S2. Performance of TCS-DL framework on carbon fibers with relatively simple morphology (*B* = 20).

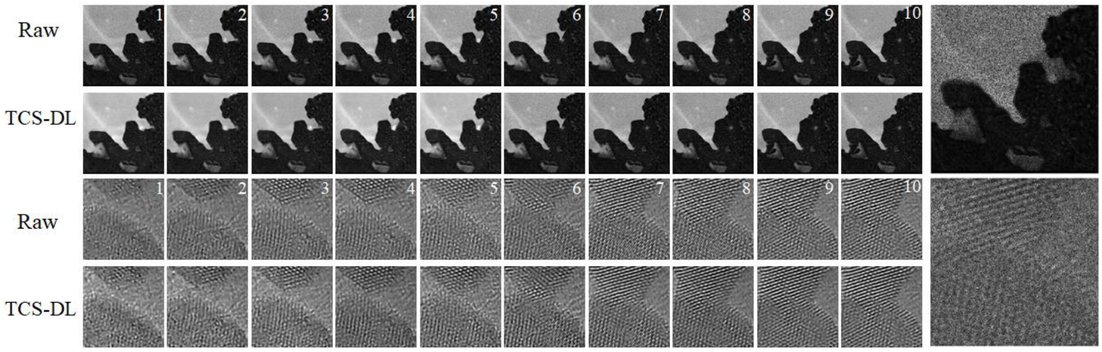

Figure S3. Additional reconstruction results with *B* = 10.

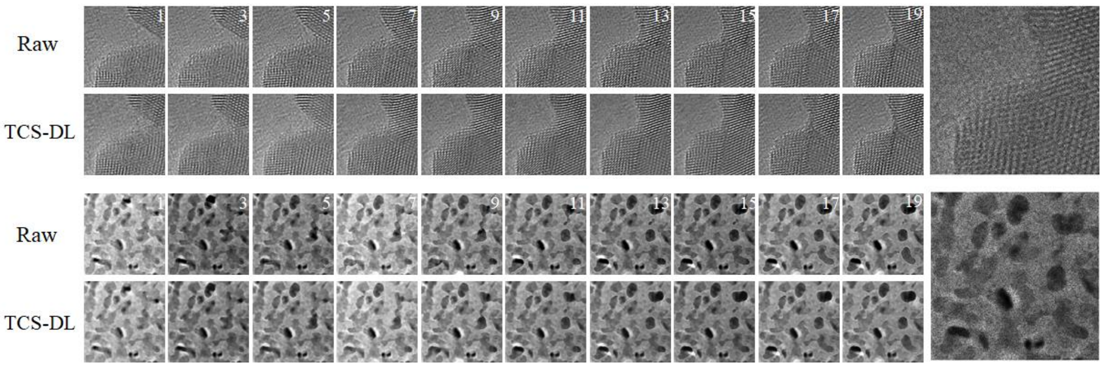

Figure S4. Additional reconstruction results with *B* = 20.

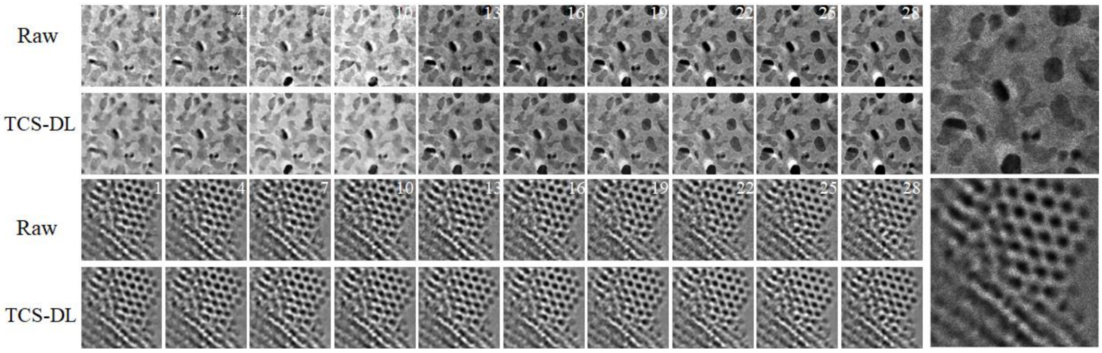

Figure S5. Additional reconstruction results with B = 30.

Raw EM images

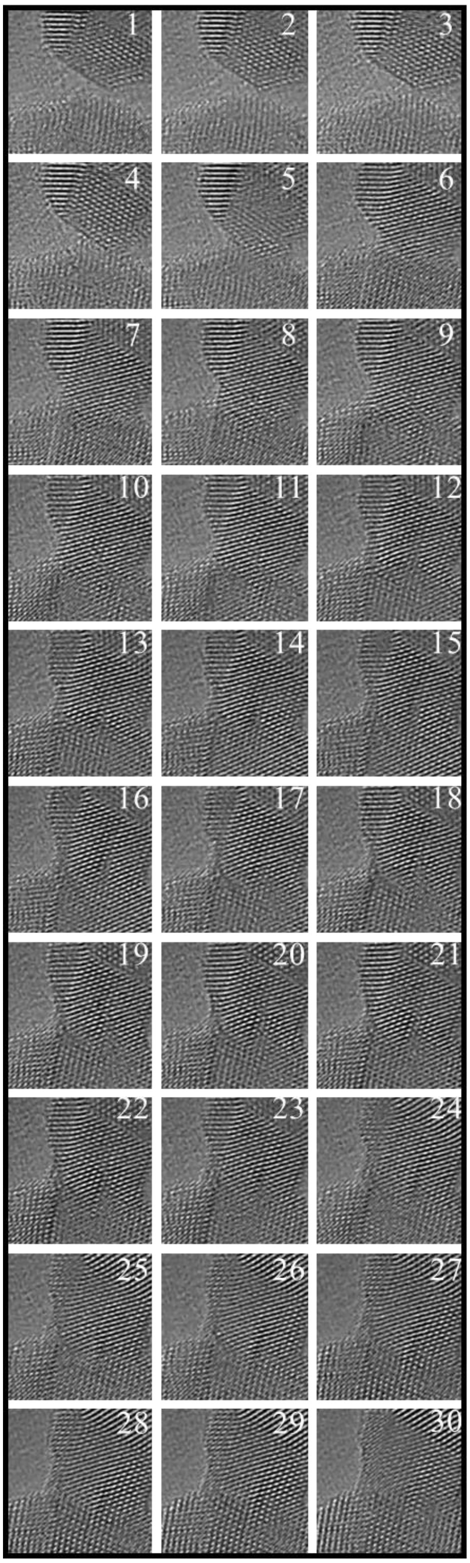

Figure S6. Raw images of Figure 2.
