# Supplementary material for: Super-compression of large electron microscopy time series by deep compressive sensing learning
Source: Patterns (N Y). 2021 Jun 24;2(7):100292. doi: 10.1016/j.patter.2021.100292 (PMC8276025; doi:10.1016/j.patter.2021.100292)
Supplement: Document S2. Article plus supplemental information [file mmc5.pdf]

Article

Patterns

Super-compression of large electron microscopy time series by deep compressive sensing learning

Graphical abstract

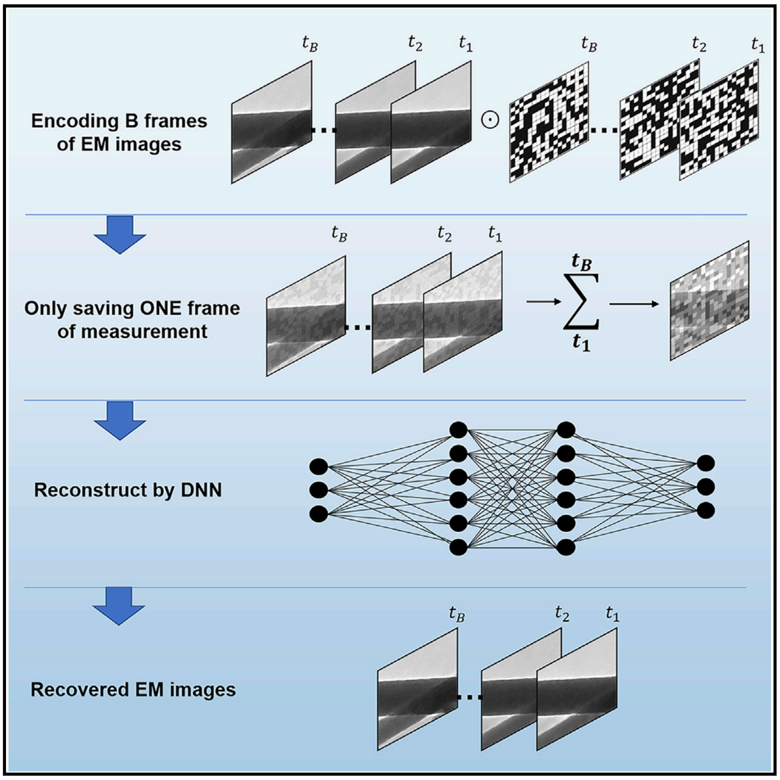

Highlights

- A novel framework, i.e., TCS-DL, has been proposed for big data compressing for EM
- The proposed TCL-DL outperforms JPEG due to the built-in denoising capability
- Considerable power, *in situ* memory, and transmission bandwidth could be saved
- The proposed TCL-DL is a novel and promising way for EM data compressing

Authors

Siming Zheng, Chunyang Wang, Xin Yuan, Huolin L. Xin

Correspondence

xyuan@bell-labs.com (X.Y.), huolinx@uci.edu (H.L.X.)

In brief

We propose a novel framework, i.e., TCL-DL, for big data compressing for EM. Owing to the significantly improved compression efficiency and built-in denoising capability of our framework over conventional JPEG compression, compressed videos with compression ratio up to 30 can be successfully reconstructed with high fidelity. Therefore, considerable (encoding) power, *in situ* memory, and transmission bandwidth are expected to be saved.

Zheng et al., 2021, Patterns 2, 100292
July 9, 2021 © 2021 The Author(s).
https://doi.org/10.1016/j.patter.2021.100292

CellPress
OPEN ACCESS

Article

Super-compression of large electron microscopy time series by deep compressive sensing learning

Siming Zheng,[1,3] Chunyang Wang,[1,3] Xin Yuan,[2,*] and Huolin L. Xin[1,4,*]
[1]Department of Physics and Astronomy, University of California, Irvine, Irvine, CA, USA
[2]Bell Labs, 600 Mountain Avenue, Murray Hill, NJ 07974, USA
[3]These authors contributed equally
[4]Lead contact
*Correspondence: xyuan@bell-labs.com (X.Y.), huolinx@uci.edu (H.L.X.)
https://doi.org/10.1016/j.patter.2021.100292

THE BIGGER PICTURE The rapid development of electron microscopy (EM) opens a new door to exploring physical sciences; however, it raises grand challenges and urgent needs for big data processing. Therefore, it is crucial to compress the EM data. But existing compression methods developed for natural images do not perform well in EM images. In this paper, by combining deep learning and temporal compressive sensing, we propose a novel compression strategy specifically for EM data processing. Owing to the improved compression efficiency and built-in denoising capability of our framework over JPEG compression, compressed videos with compression ratio of 30 can be reconstructed with high fidelity. Therefore, considerable (encoding) power, *in situ* memory, and transmission bandwidth are expected to be saved. In the future, we will strive to increase the compression ratio without reducing the reconstruction quality. And we believe our proposed EM compression method has a wide application for the EM community.

1 2 3 4 5 **Proof-of-Concept:** Data science output has been formulated, implemented, and tested for one domain/problem

SUMMARY

The development of ultrafast detectors for electron microscopy (EM) opens a new door to exploring dynamics of nanomaterials; however, it raises grand challenges for big data processing and storage. Here, we combine deep learning and temporal compressive sensing (TCS) to propose a novel EM big data compression strategy. Specifically, TCS is employed to compress sequential EM images into a single compressed measurement; an end-to-end deep learning network is leveraged to reconstruct the original images. Owing to the significantly improved compression efficiency and built-in denoising capability of the deep learning framework over conventional JPEG compression, compressed videos with a compression ratio of up to 30 can be reconstructed with high fidelity. Using this approach, considerable encoding power, memory, and transmission bandwidth can be saved, allowing it to be deployed to existing detectors. We anticipate the proposed technique will have far-reaching applications in edge computing for EM and other imaging techniques.

INTRODUCTION

Electron microscopy (EM), as one of the most powerful tools nowadays in probing materials' structure and chemistry, has extensive applications in biology, physics, chemistry, and materials science owing to its high spatial resolution and chemical sensitivity.[1] EM provides rich, directly resolved information about the structure and dynamics of phenomena, spanning from the atomic scale to micrometer scale, which are of great fundamental and practical significance to society.[2] Driven by the recent advances in computer science and electron microscopes, EM techniques, especially *in situ* transmission electron microscopy (TEM),[3–10] electron tomography,[11–22] four-dimensional scanning transmission electron microscopy (4D-STEM),[23–25] and EM image processing[26,27] become more and more dependent on big data processing and storage. In particular, due to the deployment of state-of-the-art direct electron detectors, sequential images could be generated with extraordinary frame rates up to thousands of frames per second. This, on the one hand, empowers researchers with the capability to acquire more data with an ultrahigh temporal resolution to discover new

This is an open access article under the CC BY license (http://creativecommons.org/licenses/by/4.0/).

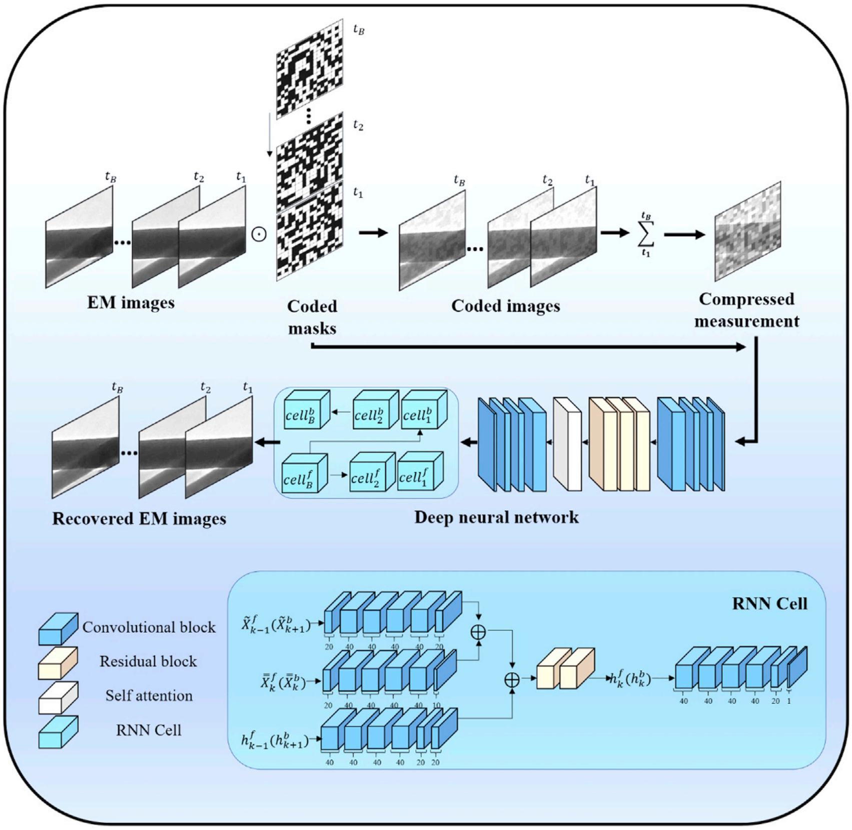

Figure 1. Workflow of the TCS-DL for big data EM

The TCS-DL consists of two parts: an encoder compressing *B* frames of EM images using a *B* random binary mask into a single compressed measurement (upper part), and a decoder using a deep neural network to reconstruct the EM images (middle part). The masks used in the encoder and the compressed measurement are first sent into a CNN to generate the first video frame. Then a forward RNN is used to generate the consequent frames. After this, a backward RNN is used to refine the frames in a reverse order to output the final EM images.

phenomena in nature. On the other hand, it poses great challenges to processing, storing, and transmitting large high-resolution EM videos or images. Compressive sensing (CS),[28–30]as an efficient signal processing technique, has been widely used in EM for data acquisition and reconstruction. It has been wildly used in capturing high-dimensional data, such as videos[31–34] and hyperspectral images.[35–40] As long as the data or signal are compressible or sparse in a certain transform domain, a measurement matrix that is not related to the transform base can be used to project the high-dimensional data obtained by the transformation onto a low-dimensional space, and, by solving an optimization problem, the original data can be reconstructed with high probability from few projections. Extensive attempts[41–47] have been made to apply CS to meet the growing demand for efficient EM data acquisition and processing; however, challenges still remain, possibly due to the following drawbacks. On one hand, the iteration-based nature makes the conventional reconstruction algorithms time consuming. On the other hand, some tasks have a higher requirement for hardware, and even the performance could still be unsatisfactory when the compression ratio is higher. Here, by combining CS and deep learning, we propose a novel encoding-decoding strategy to tackle the challenge facing big data EM. Specifically, temporal compressive sensing is first used as an encoder to compress multiple frames into a single-frame measurement with significantly reduced (on the order of 10×) bandwidth and memory requirements for data transmission and storage. An end-to-end deep learning network is then constructed to reconstruct the original image series from the single-frame measurement with extremely high speed. Peak signal-to-noise ratio (PSNR) and structural similarity (SSIM) evaluations show that the temporal compressive sensing-deep learning (TCS-DL) framework exhibits superior performance over the conventional JPEG compression method.

RESULTS AND DISCUSSION

The workflow of the proposed TCS-DL approach is summarized (Figure 1). Firstly, for the TCS compression, we consider *B* continuous video frames (*B* represents the number of EM images) captured at time slots $t_1,\ t_2,\ldots,t_B$. For each frame, we superimpose a random binary mask (coded masks) composed of 0 and 1 with the same probability. The coded images are then summed to form a single-frame measurement,[31,48] denoted by compressed measurement in the top right of Figure 1. Until now, the encoder part of the TCS-DL is finished with *B* EM images being compressed to a single-frame compressed measurement. Since the *B* -coded masks can be pre-determined and stored, the data size is reduced by *B* times. A multi-layer deep learning network is then constructed and employed as a decoder to reconstruct the original multi-frame images from the single-frame measurement. The deep neural network (DNN) is composed of a convolutional neural network (CNN) plus two recurrent neural networks (RNNs) (see details in the methods). During our experiments, the summing process in the encoder does not squeeze the dynamic range of the original image. It is purely a mathematical summation process. As we mentioned before about CS, the compressed process can be expressed as $y = \Phi x$, where y is the measurement, Φ is the compression matrix, and x are the original frames. Reconstructing x from y is an ill-conditioned problem. In this work, the optimization problem is solved by a DNN. It combines the CNN and RNN, which takes into account the correlation between adjacent frames; the learned correlation will help to reconstruct the result by maintaining the information included in the original images.

The performance of TCS-DL is tested on sequential atomic-resolution images of Au nanocrystals with complicated structures. These images, captured at full resolution (4k × 4k), show

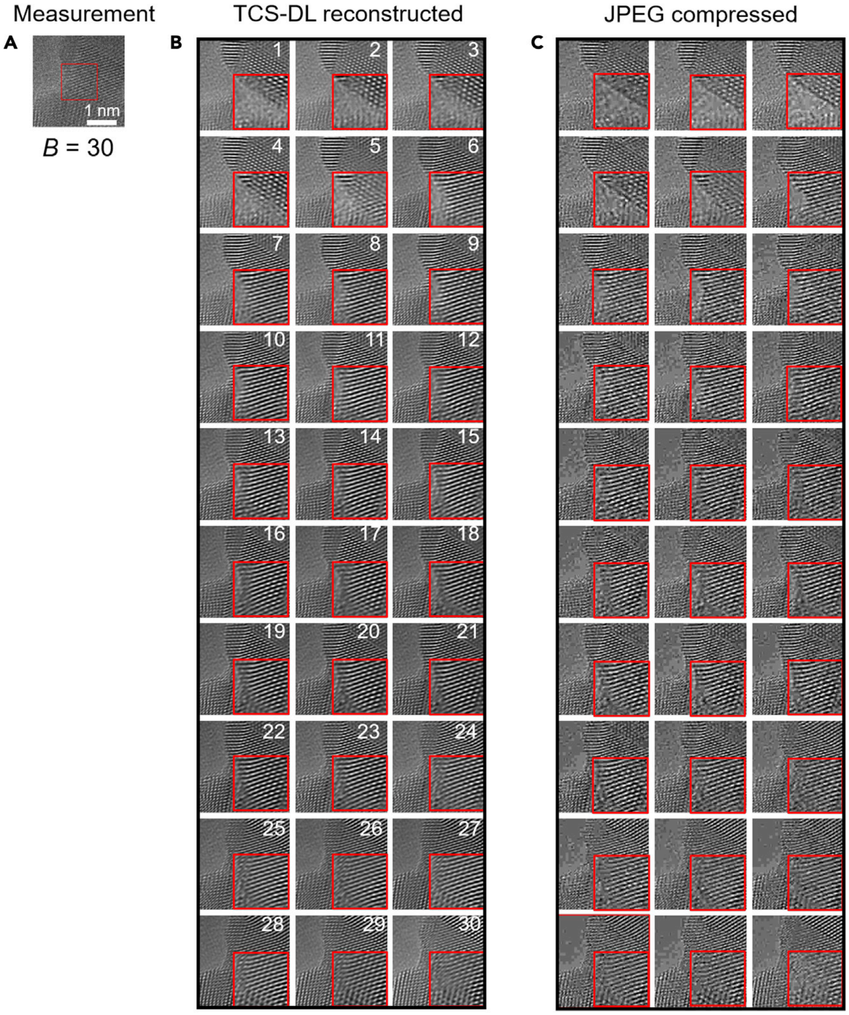

Figure 2. Performance of TCS-DL framework on *in situ* atomic-resolution EM images

(A) The single-frame compressed measurement obtained from 30 sequential images using TCS-DL.

(B) Reconstructed sequential images obtained from the compressed measurement in (A) using TCS-DL. The average PSNR is 25.91 dB compared with the denoised raw EM images.

(C) JPEG-compressed images with the total image size equal to the compressed measurement in TCS-DL. The average PSNR is 23.98 dB. Insets show magnified images of local regions (raw images in Figure S6).

the *in-situ*-sintering process of Au nanocrystals during high-resolution TEM imaging (Figure 2). The raw images are processed using the TCS-DL framework with a compression rate $B = 30$, that is, 30 sequential EM images are compressed into a single frame measurement (Figure 2A) and then reconstructed (Figure 2B). As a comparison, JPEG compression is also performed on the raw images (Figure 2C). Note that, to keep the memory consumption to store the images the same as that using TCS-DL, the size of the JPEG-compressed image is kept as $1/B$ of the original image size. The results show that obvious artifacts and noises are observed in the JPEG-compressed images. In sharp contrast, the reconstructed EM images using TCS-DL show a clean background, fine details, and sharp edges. To quantitatively evaluate the performances of TCS-DL and JPEG, we calculate the PSNR and SSIM of both JPEG-compressed images and TCS-DL reconstructed images compared with denoised raw images. Note that these raw images are usually noisy and a denoising algorithm[49,50] can be applied. In this paper, we use BM3D for denoising. However, this will incurs power and computation costs. Therefore, in this work, both JPEG and TCS-DL are performed directly on the raw images. The average PSNR for JPEG compression and TCS-DL is 23.98 and 25.91 dB, respectively. The PSNR improvement of 1.93 dB indicates an evidently improved performance of TCS-DL over JPEG, which is in good agreement with the enhanced image quality using TCS-DL (Figure 2B) compared with JPEG compression (Figure 2C).

A more detailed comparison is made on representative selected frames as shown in Figure 3. The reconstructed images show enhanced surface atomic details (indicated by the arrows in Figures 3B and 3H) compared with that in the raw image (Figure 3A). However, in sharp contrast, the JPEG-compressed image shows severely deteriorated atomic details, especially at the crystal surface, e.g., the edge blurring as indicated by the arrows (Figures 3C and 3I). Fast Fourier transform (FFT) is further performed on the raw image as well as that processed using TCS-DL and JPEG compression (Figures 3D and 3F). The result shows that the high-frequency information (e.g., the (004) plane of the Au nanocrystal) in the raw image is well retained in the reconstructed image using TCS-DL. In contrast, the FFT of the JPEG-compressed image shows deteriorated resolution (indicated by the unresolved (004) plane of the Au nanocrystal) compared with the raw image and reconstructed image using the TCS-DL framework. The above results reveal that, distinct from the conventional JPEG, which introduces information loss during compression, the TCS-DL framework is capable of recovering the detailed information in the raw data. In addition, the built-in denoising capability of TCS-DL could further slightly improve the quality of raw images.

Aside from atomic-resolution images of ultrasmall nano-objects, we further tested the performance of the TCS-DL framework on objects with a much larger feature size in a large field of view. Figure 4 shows sequential EM images of two carbon fibers during *in situ* deformation. The raw images are processed using TCS-DL and JPEG compression, respectively. The TCS-DL reconstructed images show a clean background as well as a sharp edge, which thereby enables accurate tracking of the motions of the fibers (Figure 4C). In contrast, the JPEG-compressed images inherit the noises in the raw images, similar to

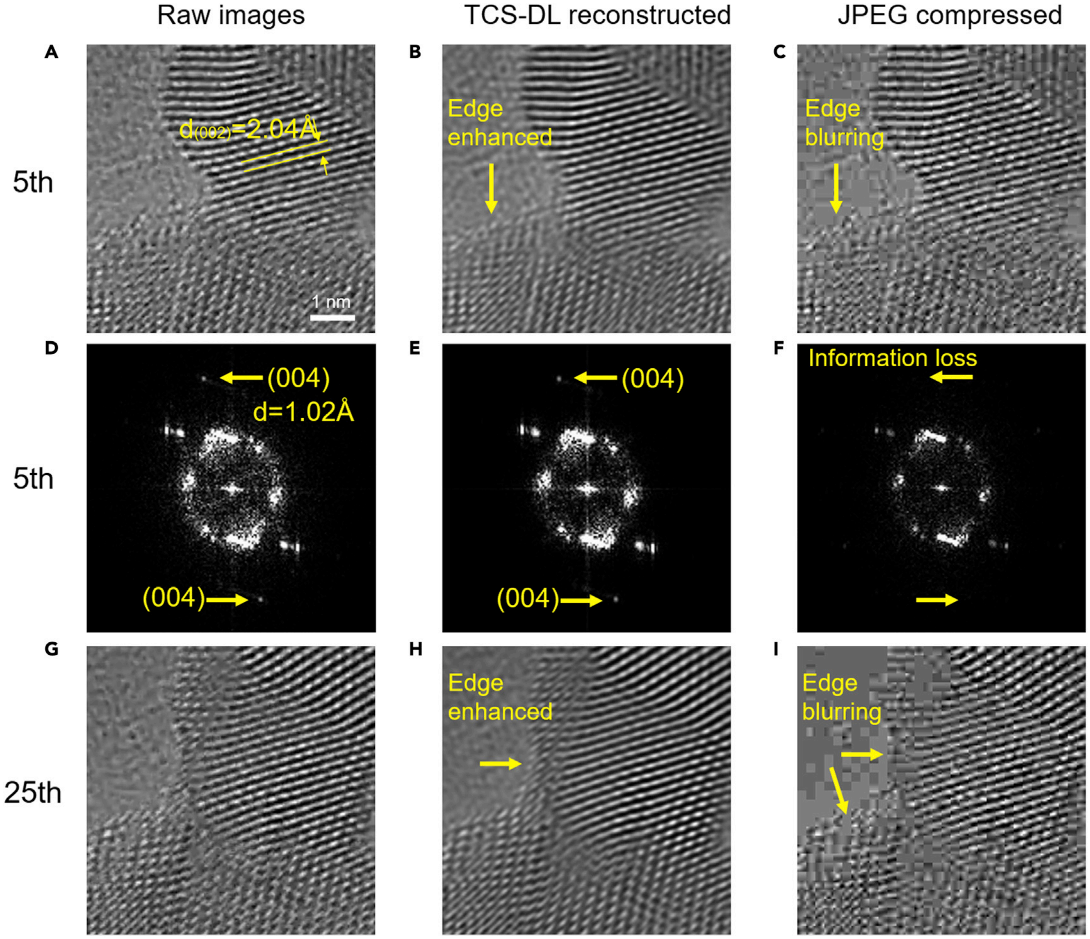

Figure 3. Comparison of selected atomic-resolution images obtained using TCS-DL and JPEG compression in Figure 2
(A–C) 5th raw images, reconstructed images using TCS-DL and JPEG compression.
(D–F) Fast Fourier transforms of the images in (A–C). Bragg spots corresponding to the (004) planes are indicated by the arrows in (D and E). Information loss is identified in the JPEG-compressed image (F) as the Bragg spots corresponding to (004) planes are not resolved.
(G–I) 25th raw images, reconstructed images using TCS-DL and JPEG compression. Compared to the raw image (G), more surface details are observed in the TCS-DL-processed image (H) due to the edge enhancement. In contrast, deteriorated image quality such as edge blurring is identified in the JPEG-compressed image (I).

that in atomic-resolution images as shown in Figures 2 and 3. It is worth noting that, due to the relatively simple morphology of the carbon fibers compared with Au nanocrystals, the PSNR of both JPEG compression and TCS-DL framework are evidently higher than that of the atomic-resolution images, as shown in Figures 2 and 3. Yet, the TCS-DL framework still outperforms JPEG compression with a considerable PSNR improvement of approximately 3 dB, from 32.38 dB (JPEG compression) to 35.41 dB (TCS-DL). Similar performance improvement is also obtained for EM images reconstructed from TCS-DL at $B = 20$ (Figure S1). Results of different compression ratios are shown in Video S1. We also present the results of different video compression algorithms in Videos S2 and S3.

PSNR and SSIM are calculated and summarized to quantitatively compare the performance of the TCS-DL framework and JPEG compression. As shown in Figure 5, we conduct both TCS-DL and JPEG compression at different compression ratios from $B = 10$ to $B = 30$. PSNR, which is a ratio between the maximum possible power of a signal and the power of corrupting noise that affects the fidelity of its representation, is mainly used to evaluate the performance of reconstructed EM images using different compression methods. SSIM is used for measuring the similarity between the reconstructed EM images and the corresponding ground truth. Clearly, TCS-DL has higher PSNR and SSIM, which means that it can get better performance, especially when the raw images are noisier (Figures 2 and 4) and the compression ratio (Figure 2) is high, because our framework can reduce the background noise while JPEG produces obvious artifacts when the compression ratio is high. What needs to be mentioned is that the reconstruction process in this work utilizes both CNN and RNN structures, where RNN is mainly used to learn the correlation between adjacent frames. If the frames are obviously incoherent, the reconstruction result would be vulnerable. In most instances, the frames in the *in situ* TEM movie are coherent. We can utilize our compression method under the condition just mentioned. But at the edge, when the content in

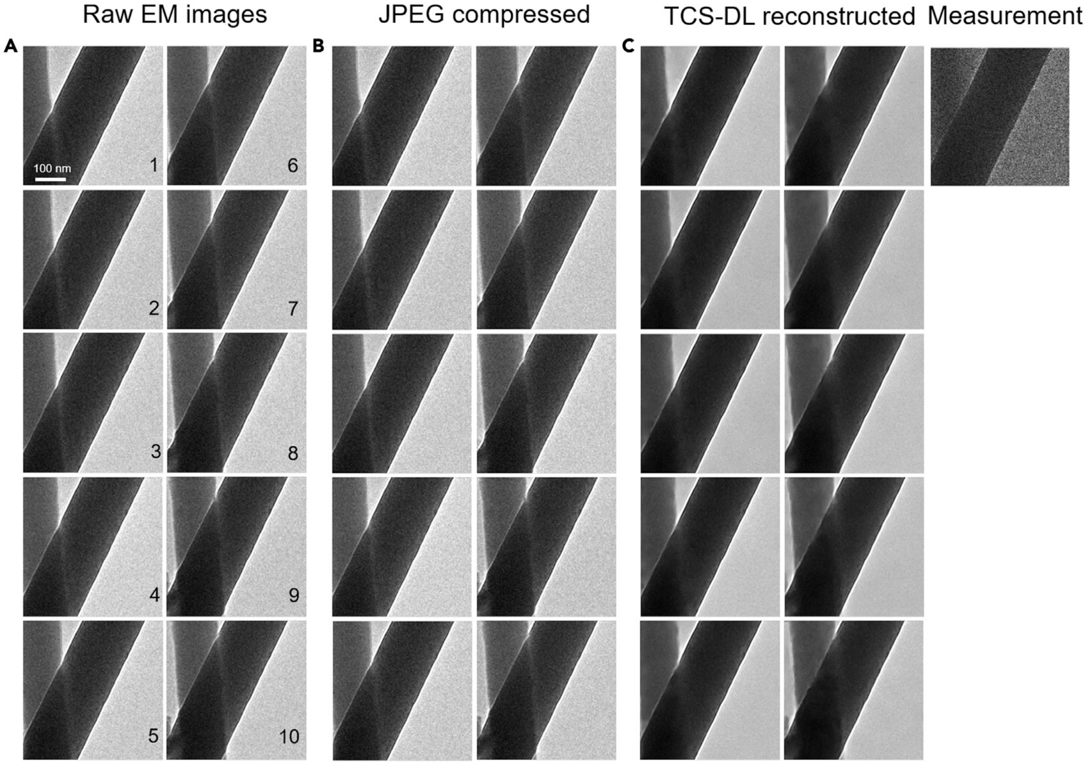

Figure 4. Performance of the TCS-DL framework on carbon fibers with relatively simple morphology ($B = 10$)

(A) Raw images of the carbon fibers during *in situ* deformation.

(B) JPEG-compressed images with the total image size equal to the compressed measurement in TCS-DL. The average PSNR is 32.38 dB compared with the denoised raw images.

(C) Reconstructed images using the TCS-DL framework. The average PSNR is 35.41 dB. Note that the JPEG-compressed images preserve the noise in the raw images (additional results in Figures S3–S5).

the receptive field changes suddenly, we do not use this method to compress the incoherent frames. In future work, we can automatically detect the sudden change and choose the corresponding compression method. If the receptive field does not change frequently, which means that the coherent frame number is sufficiently greater than the compression ratio B, TCS-DL can achieve a generally impressive result.

The training loss and validation loss during the learning process with $B = 10$ are shown in Figure 6. As we can see, the validation loss gradually converges as the training process progresses. Because we have sufficient data and we stop the training at the appropriate epoch, the validation loss curve does not show a V-shaped trend,which means it is not overfitted.

Conclusion

In conclusion, by combining deep learning and temporal compressive sensing, a novel encoding-decoding framework, i.e., TCS-DL, has been proposed for big data compression/decompression for EM. Owing to the significantly improved compression efficiency and built-in denoising capability of the TCS-DL framework over the conventional JPEG compression method, compressed EM time series with a high compression ratio can be successfully reconstructed with high fidelity by the TCS-DL framework from a single measurement. Our work, by offering a universally applicable super-compression strategy for EM data compression, opens the possibility for EM big data storage, processing, and transmission at the expense of significantly reduced power, *in situ* memory, and transmission bandwidth.

EXPERIMENTAL PROCEDURES

Resource availability

Lead contact

Further information and requests for resources and reagents should be directed to and will be fulfilled by the lead contact, Huolin L. Xin (huolinx@uci.edu).

Materials availability

This study did not generate new unique reagents.

Data and code availability

The original data within this paperis available from our website http://temimagenet.ps.uci.edu/. The code used in this work is available at https://github.com/xinhuolin/TCS-DL.

Methods

We first derive the mathematical model of TCS-DL and then present the details of the DNN for reconstruction.

Let $X_1, \ldots, X_B$ denote the B frames of the EM images being coded with masks $C_1, \ldots, C_B$. Each frame is of spatial size $N_x \times N_y$ pixels. From Figure 1, the coded measurement can be expressed as

$$Y = \sum_{b=1}^{B} X_b \odot C_b. \quad \text{(Equation 1)}$$

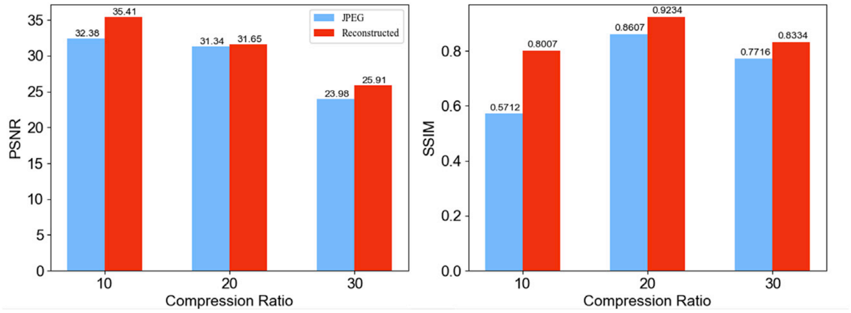

Figure 5. Average PSNR (in dB), SSIM of TCS-DL and JPEG at different compression ratios

Denoised raw EM images are used as a baseline for calculation of the PSNR and SSIM. The results are based on the data in Figures 3, 4, and S2.

Here, $\odot$ denotes the element-wise product. Note that since the masks are composed of 0 and 1, there is no multiplication in Equation 1. We only need to index the "1" elements in the corresponding frames and sum across the B frames to get the compressed measurement Y. Therefore, our proposed encoder is ultra-simple. Indeed, only Equation 1 will finish the encoder and the complexity is of $O(1)$.

The decoder aims to estimate the EM images, which can be denoised based on the previous analysis, from Y, given masks. As shown in Figure 1, we use a CNN and two RNNs to perform the reconstruction.[51,52]

The more information RNN takes as input, the better results we get. Therefore, the first frame for RNN with as much visual information as possible is required. To this purpose, we utilize a CNN with 12 layers to reconstruct the first input frame. We introduce normalized measurement $\overline{Y}$

$$\overline{Y} = Y \oslash \left(\sum_{k=1}^{B} C_K\right) \quad \text{(Equation 2)}$$

as input component of CNN. Where $\oslash$ denotes the matrix division. We concatenate $\overline{Y}$, $\overline{Y}\odot C_1$, $\overline{Y}\odot C_2, \ldots \overline{Y}\odot C_B$ along the third dimension. The concatenation result is then fed into the CNN which consists of two four-layer CNNs, one three-layer residual block,[53] and one self-attention block.[54] Then, the first frame $\tilde{X}^f_1$ for RNN is generated. It is worth noting that $\overline{Y}\odot C_K$ is to approximate the real coded frames.

After getting $\tilde{X}^f_1$, we introduce two RNNs with different directions to reconstruct the frames. As shown in Figure 1, the input of each RNN cell consists of three different parts. The first part is got from the former RNN cell's output $\tilde{X}^f_{k-1}(\tilde{X}^b_{k+1})$ except the first $\tilde{X}^f_1$, which is obtained from the CNN. The second part $\overline{\overline{X}}^f_k(\overline{\overline{X}}^b_k)$ is acquired by

$$\overline{\overline{X}}^f_k = \overline{Y} \oplus \left(Y - \sum_{t=1}^{k-1} C_k \odot \tilde{X}^f_t - \sum_{t=k+1}^{b} C_t \odot \overline{Y}\right), \quad \text{(Equation 3)}$$

where $\oplus$ denotes concatenation along the third dimension which has the same meaning in Figure 1. The second item in the right part of Equation 3 could be considered to be an approximation of $C_k \odot X_k$ to some extent. The third part $h^f_{k-1}(h^b_{k+1})$ is hidden units obtained by former RNN cell except the first one h^f_1 initialized by zero. The three input parts are separately fed into three sub-CNNs. After the process of concatenation, there are two residual blocks, which outputs the new hidden unit fed to another sub-CNN. Then, each cell outputs the reconstructed frame. Noting that forward and backward RNNs have the same structure, but they do not share the parameters. Besides, the second input part of the backward RNN cell is different from the forward RNN cell

$$\overline{\overline{X}}^b_k = \overline{Y} \oplus \left(Y - \sum_{t=B, t\neq k}^{1} C_k \odot \tilde{X}^f_t\right), \quad \text{(Equation 4)}$$

which uses reconstruction $\tilde{X}^f_k$ from forward RNN instead of $\overline{Y}$. Another difference is that the initial hidden unit for backward RNN is not set to zero but h^f_B which contains more information.

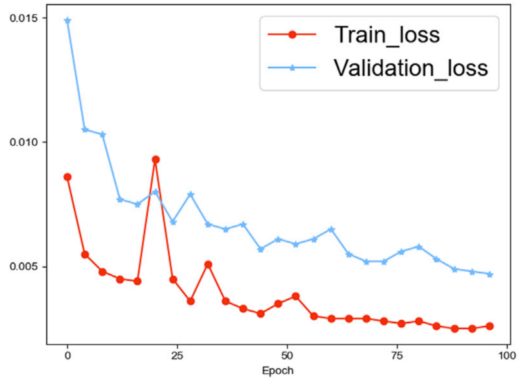

Figure 6. The training loss and validation loss during the learning process with B = 10

The validation loss gradually converges as the training process progresses.

SUPPLEMENTAL INFORMATION

Supplemental information can be found online at https://doi.org/10.1016/j.patter.2021.100292.

ACKNOWLEDGMENTS

This material is based on work supported by the Early Career Research Program, Materials Science and Engineering Divisions, Office of Basic Energy Sciences of the U.S. Department of Energy, under award no. DE-SC0021204 (program manager Dr. Jane Zhu). S.Z.'s effort on this project was supported by H.L.X.'s startup funding.

AUTHOR CONTRIBUTIONS

H.L.X. and X.Y. conceived the idea. All authors designed and conducted the study. All authors wrote the manuscript.

DECLARATION OF INTERESTS

The authors declare no competing interests. The third author, Xin Yuan, is an employee of Nokia.

Received: February 25, 2021
Revised: April 21, 2021
Accepted: May 26, 2021
Published: June 24, 2021

REFERENCES

1. Ruska, E. (1987). The development of the electron microscope and of electron microscopy. Rev. Mod. Phys. *59*, 627–638. https://doi.org/10.1103/RevModPhys.59.627.

CellPress
OPEN ACCESS

2. Spurgeon, S.R., Ophus, C., Jones, L., Petford-Long, A., Kalinin, S.V., Olszta, M.J., Dunin-Borkowski, R.E., Salmon, N., Hattar, K., Yang, W.D., et al. (2020). Towards data-driven next-generation transmission electron microscopy. Nat. Mater. https://doi.org/10.1038/s41563-020-00833-z.
3. Wang, C., Han, L., Zhang, R., Cheng, H., Mu, L., Kisslinger, K., et al. (2021). Resolving atomic-scale phase transformation and oxygen loss mechanism in ultrahigh-nickel layered cathodes for cobalt-free lithium-ion batteries. Matter *4* (6), 2013–2026. https://doi.org/10.1016/j.matt.2021.03.012.
4. Song, M., Zhou, G., Lu, N., Lee, J., Nakouzi, E., Wang, H., and Li, D. (2020). Oriented attachment induces fivefold twins by forming and decomposing high-energy grain boundaries. Science *367*, 40–45. https://doi.org/10.1126/science.aax6511.
5. Chen, Y., Wang, Z., Li, X., Yao, X., Wang, C., Li, Y., Xue, W., Yu, D., Kim, S.Y., Yang, F., et al. (2020). Li metal deposition and stripping in a solid-state battery via Coble creep. Nature *578*, 251–255. https://doi.org/10.1038/s41586-020-1972-y.
6. He, S., Wang, C., Qi, L., Ye, H., and Du, K. (2020). In situ observation of twin-assisted grain growth in nanometer-scaled metal. Micron *131*, 102825. https://doi.org/10.1016/j.micron.2020.102825.
7. Sang, X., Xie, Y., Yilmaz, D.E., Lotfi, R., Alhabeb, M., Ostadhossein, A., Anasori, B., Sun, W., Li, X., Xiao, K., et al. (2018). In situ atomistic insight into the growth mechanisms of single layer 2D transition metal carbides. Nat. Commun. *9*, 2266. https://doi.org/10.1038/s41467-018-04610-0.
8. Liao, H.-G., Zherebetskyy, D., Xin, H., Czarnik, C., Ercius, P., Elmlund, H., Pan, M., Wang, L.-W., and Zheng, H. (2014). Facet development during platinum nanocube growth. Science *345*, 916–919. https://doi.org/10.1126/science.1253149.
9. Wang, X., Wang, C., Chen, C., Duan, H., and Du, K. (2019). Free-standing monatomic thick two-dimensional gold. Nano Lett. *19*, 4560–4566. https://doi.org/10.1021/acs.nanolett.9b01494.
10. Wang, C., Du, K., Song, K., Ye, X., Qi, L., He, S., Tang, D., Lu, N., Jin, H., Li, F., and Ye, H. (2018). Size-dependent grain-boundary structure with improved conductive and mechanical stabilities in sub-10-nm gold crystals. Phys. Rev. Lett. *120*, 186102. https://doi.org/10.1103/PhysRevLett.120.186102.
11. Zhou, J., Yang, Y., Yang, Y., Kim, D.S., Yuan, A., Tian, X., Ophus, C., Sun, F., Schmid, A.K., Nathanson, M., et al. (2019). Observing crystal nucleation in four dimensions using atomic electron tomography. Nature *570*, 500–503. https://doi.org/10.1038/s41586-019-1317-x.
12. Wang, C., Duan, H., Chen, C., Wu, P., Qi, D., Ye, H., Jin, H.-J., Xin, H.L., and Du, K. (2020). Three-dimensional atomic structure of grain boundaries resolved by atomic-resolution electron tomography. Matter *3*, 1999–2011. https://doi.org/10.1016/j.matt.2020.09.003.
13. Miao, J., Ercius, P., and Billinge, S.J. (2016). Atomic electron tomography: 3D structures without crystals. Science *353*. https://doi.org/10.1126/science.aaf2157.
14. Eggeman, A.S., Krakow, R., and Midgley, P.A. (2015). Scanning precession electron tomography for three-dimensional nanoscale orientation imaging and crystallographic analysis. Nat. Commun. *6*, 7267. https://doi.org/10.1038/ncomms8267.
15. Albrecht, W., and Bals, S. (2020). Fast electron tomography for nanomaterials. J. Phys. Chem. C *124*, 27276–27286. https://doi.org/10.1021/acs.jpcc.0c08939.
16. Kim, B.H., Heo, J., Kim, S., Reboul, C.F., Chun, H., Kang, D., Bae, H., Hyun, H., Lim, J., Lee, H., et al. (2020). Critical differences in 3D atomic structure of individual ligand-protected nanocrystals in solution. Science *368*, 60–67. https://doi.org/10.1126/science.aax3233.
17. Liao, M., Cao, E., Julius, D., and Cheng, Y. (2013). Structure of the TRPV1 ion channel determined by electron cryo-microscopy. Nature *504*, 107. https://doi.org/10.1038/nature12822.
18. Ding, G., Liu, Y., Zhang, R., and Xin, H.L. (2019). A joint deep learning model to recover information and reduce artifacts in missing-wedge sinograms for electron tomography and beyond. Sci. Rep. *9*, 12803. https://doi.org/10.1038/s41598-019-49267-x.
19. Wang, C., Ding, G., Liu, Y., and Xin, H.L. (2020). 0.7 Å resolution electron tomography enabled by deep-learning-aided information recovery. Adv. Intell. Syst. *2*, 2000152. https://doi.org/10.1002/aisy.202000152.
20. Han, L., Meng, Q., Wang, D., Zhu, Y., Wang, J., Du, X., Stach, E.A., and Xin, H.L. (2016). Interrogation of bimetallic particle oxidation in three dimensions at the nanoscale. Nat. Commun. *7*, 13335. https://doi.org/10.1038/ncomms13335.
21. Wang, D., He, H., Han, L., Lin, R., Wang, J., Wu, Z., Liu, H., and Xin, H.L. (2016). Three-dimensional hollow-structured binary oxide particles as an advanced anode material for high-rate and long cycle life lithium-ion batteries. Nano Energy *20*, 212–220. https://doi.org/10.1016/j.nanoen.2015.12.019.
22. Lin, R., Bak, S.M., Shin, Y., Zhang, R., Wang, C., Kisslinger, K., Ge, M., Huang, X., Shadike, Z., Pattammattel, A., et al. (2021). Hierarchical nickel valence gradient stabilizes high-nickel content layered cathode materials. Nat. Commun. *12*, 2350. https://doi.org/10.1038/s41467-021-22635-w.
23. Jiang, Y., Chen, Z., Han, Y., Deb, P., Gao, H., Xie, S., Purohit, P., Tate, M.W., Park, J., Gruner, S.M., et al. (2018). Electron ptychography of 2D materials to deep sub-angstrom resolution. Nature *559*, 343–349. https://doi.org/10.1038/s41586-018-0298-5.
24. Ophus, C., Ercius, P., Sarahan, M., Czarnik, C., and Ciston, J. (2014). Recording and using 4D-STEM datasets in materials science. Microsc. Microanalysis *20*, 62–63. https://doi.org/10.1017/s1431927614002037.
25. Gao, W., Addiego, C., Wang, H., Yan, X., Hou, Y., Ji, D., Heikes, C., Zhang, Y., Li, L., Huyan, H., et al. (2019). Real-space charge-density imaging with sub-angstrom resolution by four-dimensional electron microscopy. Nature *575*, 480–484. https://doi.org/10.1038/s41586-019-1649-6.
26. Lin, R., Zhang, R., Wang, C., Yang, X.Q., and Xin, H.L. (2021). TEMImageNet training library and AtomSegNet deep-learning models for high-precision atom segmentation, localization, denoising, and deblurring of atomic-resolution images. Sci. Rep. *11*, 5386. https://doi.org/10.1038/s41598-021-84499-w.
27. Wang, Chunyang, Zhang, Rui, Kisslinger, Kim, and Xin, Huolin L. (2021). Atomic-Scale Observation of O1 Faulted Phase-Induced Deactivation of $LiNiO_2$ at High Voltage. Nano Letters *21*, 3657–3663. https://doi.org/10.1021/acs.nanolett.1c00862.
28. Candès, E.J., Romberg, J., and Tao, T. (2006). Robust uncertainty principles: exact signal reconstruction from highly incomplete frequency information. IEEE Trans. Inf. Theor. *52*, 489–509.
29. Candès, E.J., and Wakin, M.B. (2008). An introduction to compressive sampling. IEEE Signal. Process. Mag. *25*, 21–30.
30. Donoho, D.L. (2006). Compressed sensing. IEEE Trans. Inf. Theor. *52*, 1289–1306.
31. Hitomi, Y., Gu, J., Gupta, M., Mitsunaga, T., and Nayar, S.K. (2011). Video from a Single Coded Exposure Photograph Using a Learned Over-complete Dictionary (IEEE), pp. 287–294.
32. Reddy, D., Veeraraghavan, A., and Chellappa, R. (2011). P2C2: Programmable Pixel Compressive Camera for High Speed Imaging (IEEE), pp. 329–336.
33. Yuan, X., and Pang, S. (2016). Compressive Video Microscope via Structured Illumination (IEEE), pp. 1589–1593.
34. Yuan, X., and Pang, S. (2016). Structured illumination temporal compressive microscopy. Biomed. Opt. Express *7*, 746–758.
35. Gehm, M.E., John, R., Brady, D.J., Willett, R.M., and Schulz, T.J. (2007). Single-shot compressive spectral imaging with a dual-disperser architecture. Opt. Express *15*, 14013–14027.
36. Wagadarikar, A., John, R., Willett, R., and Brady, D. (2008). Single disperser design for coded aperture snapshot spectral imaging. Appl. Opt. *47*, B44–B51.
37. Wagadarikar, A.A., Pitsianis, N.P., Sun, X., and Brady, D.J. (2009). Video rate spectral imaging using a coded aperture snapshot spectral imager. Opt. Express *17*, 6368–6388.
38. Tsai, T.-H., Llull, P., Yuan, X., Carin, L., and Brady, D.J. (2015). Spectral-temporal compressive imaging. Opt. Lett. *40*, 4054–4057.

39. Zheng, S., Liu, Y., Meng, Z., Qiao, M., Tong, Z., Yang, X., Han, S., and Yuan, X. (2021). Deep plug-and-play priors for spectral snapshot compressive imaging. Photon. Res. *9*, B18–B29.
40. Yuan, X., Brady, D.J., and Katsaggelos, A.K. (2021). Snapshot compressive imaging: theory, algorithms, and applications. IEEE Signal. Process. Mag. *38*, 65–88.
41. Foucart, S., and Rauhut, H. (2013). An invitation to compressive sensing. In A Mathematical Introduction to Compressive Sensing (Springer), pp. 1–39.
42. Saghi, Z., Holland, D.J., Leary, R., Falqui, A., Bertoni, G., Sederman, A.J., Gladden, L.F., and Midgley, P.A. (2011). Three-dimensional morphology of iron oxide nanoparticles with reactive concave surfaces. A compressed sensing-electron tomography (CS-ET) approach. Nano Lett. *11*, 4666–4673.
43. Stevens, A., Yang, H., Carin, L., Arslan, I., and Browning, N.D. (2014). The potential for Bayesian compressive sensing to significantly reduce electron dose in high-resolution STEM images. Microscopy *63*, 41–51.
44. Stevens, A., Luzi, L., Yang, H., Kovarik, L., Mehdi, B., Liyu, A., Gehm, M., and Browning, N. (2018). A sub-sampled approach to extremely low-dose STEM. Appl. Phys. Lett. *112*, 043104.
45. Van den Broek, W., Reed, B.W., Béché, A., Velazco, A., Verbeeck, J., and Koch, C.T. (2019). Various compressed sensing setups evaluated against Shannon sampling under constraint of constant illumination. IEEE Trans. Comput. Imaging *5*, 502–514.
46. Reed, B.W., Moghadam, A., Bloom, R., Park, S., Monterrosa, A., Price, P., Barr, C., Briggs, S., Hattar, K., and McKeown, J. (2019). Electrostatic subframing and compressive-sensing video in transmission electron microscopy. Struct. Dyn. *6*, 054303.
47. Reed, B.W., Park, S.T., Bloom, R.S., and Masiel, D.J. (2017). Compressively sensed video acquisition in transmission electron microscopy. Microsc. Microanalysis *23*, 84–85.
48. Liu, Y., Yuan, X., Suo, J., Brady, D.J., and Dai, Q. (2018). Rank minimization for snapshot compressive imaging. IEEE Trans. pattern Anal. Mach. Intellig. *41*, 2990–3006.
49. Yoshizawa, T., Hirobayashi, S., and Misawa, T. (2011). Noise reduction for periodic signals using high-resolution frequency analysis. EURASIP J. Audio Speech Music Process. *2011*, 5.
50. Dabov, K., Foi, A., Katkovnik, V., and Egiazarian, K. (2006). Image Denoising with Block-Matching and 3D Filtering (International Society for Optics and Photonics), p. 606414.
51. Cheng, Z., Lu, R., Wang, Z., Zhang, H., Chen, B., Meng, Z., and Yuan, X. (2020). BIRNAT: Bidirectional Recurrent Neural Networks with Adversarial Training for Video Snapshot Compressive Imaging (Springer), pp. 258–275.
52. Schuster, M., and Paliwal, K.K. (1997). Bidirectional recurrent neural networks. IEEE Transactions Signal. Process. *45*, 2673–2681.
53. He, K., Zhang, X., Ren, S., and Sun, J. (2016). Deep residual learning for image recognition. Proceedings of the IEEE conference on computer vision and pattern recognition, 770–778. https://openaccess.thecvf.com/content_cvpr_2016/html/He_Deep_Residual_Learning_CVPR_2016_paper.html.
54. Vaswani, A., Shazeer, N., Parmar, N., Uszkoreit, J., Jones, L., Gomez, A.N., Kaiser, Ł., and Polosukhin, I. (2017). Attention Is All You Need. arXiv, 1706.03762. https://arxiv.org/abs/1706.03762.

Patterns, Volume *2*

Supplemental information

Super-compression of large electron microscopy time series by deep compressive sensing learning

Siming Zheng, Chunyang Wang, Xin Yuan, and Huolin L. Xin

SUPPLEMENTAL EXPERIMENTAL PROCEDURES

Training Details

Our reconstruction network is trained on the data captured by ourself with the pixel resolution of 2048*2048 and published video data on the internet. We cropped patches of size 256x*256 with 3 different compression ratios (CR). The respective number of patches for each network with B = 10, 20, and 30 is 3000, 3500 and 4000. We use PyTorch for implementation and Adam as the optimizer. The total number of training epochs is respectively set to 100, 150, and 250 with a learning rate of $5*10^{-4}$ which decays 10% every 10 epochs before epoch 100 and decays 5% every 20 epochs after epoch 100. Training the network with B=10 took approximately 5 days which is run on a Nvidia GTX 1080 Ti GPU with 11GB RAM. The networks with B = 20, 30 are all run on the NVIDIA RTX 8000 GPU with 48GB RAM which take respectively approximately one and three weeks.

Decoding Time

As shown in Fig. S1, TCS-DL can reconstruct EM images efficiently. We also compare with another deep learning reconstruction method U-net only with B = 10. It is a little bit faster than TCS-DL, but it does not take adjacent frames' correlation into consideration and the model is fixed so that it can not get as satisfactory reconstruction results as TCS-DL when the compression ratio increases. As we can see, the decoding time increases as the compression ratio gets higher for the reason that TCS-DL's parameters increase as the compression ratio gets higher. In spite of the decoding time is got at the resolution of 256*256, TCS-DL can be paralleled on multiple GPUs which only has negligible latency, we can reconstruct EM images with larger resolution in approximately the same time. Besides we could apply TensorRT to speed up TCS-DL by inference optimization in practice.

Data Availability

The datasets and source codes to reproduce the results are available from the corresponding author on reasonable request.

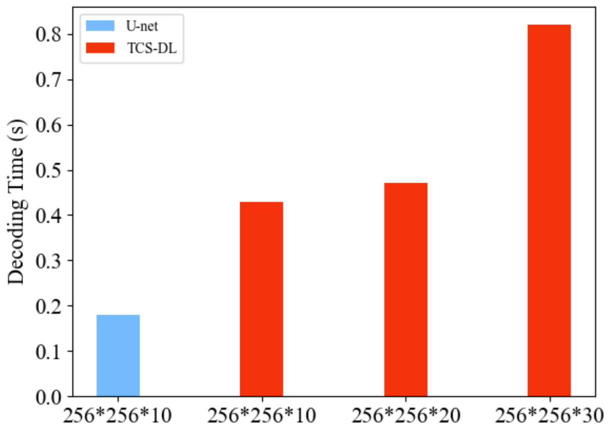

Figure S1. Decoding time of TCS-DL with *B* = 10, 20, and 30 and U-net with *B* = 10.

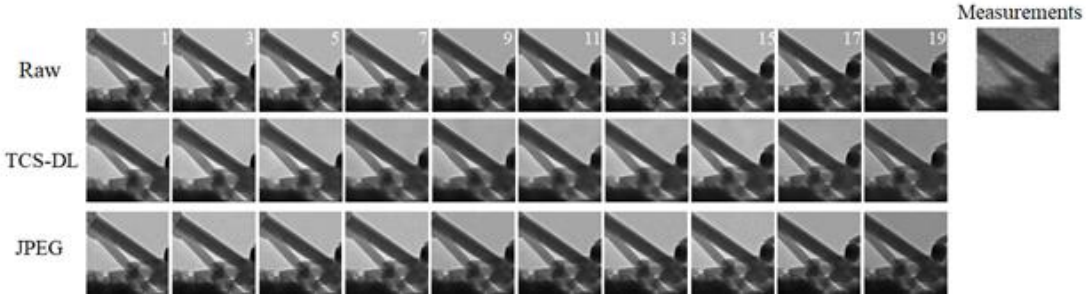

Figure S2. Performance of TCS-DL framework on carbon fibers with relatively simple morphology (*B* = 20).

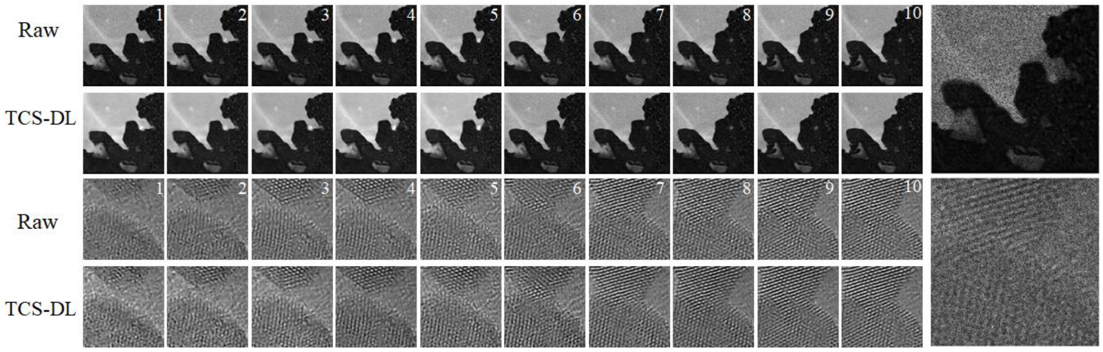

Figure S3. Additional reconstruction results with *B* = 10.

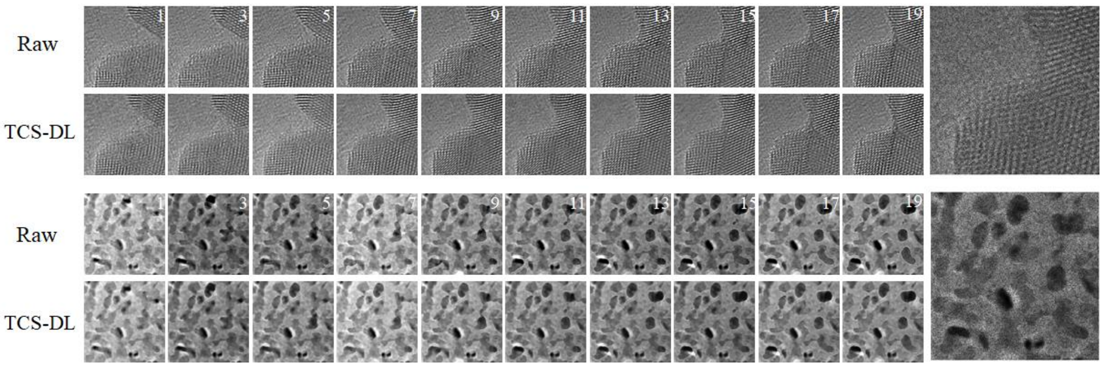

Figure S4. Additional reconstruction results with *B* = 20.

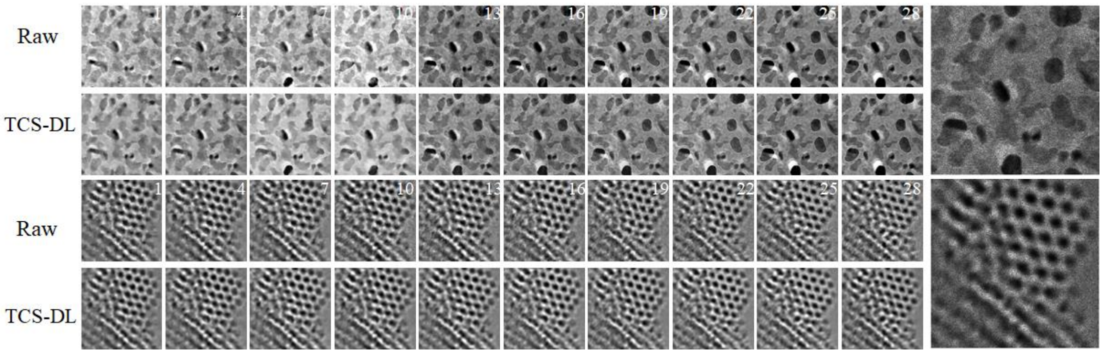

Figure S5. Additional reconstruction results with *B* = 30.

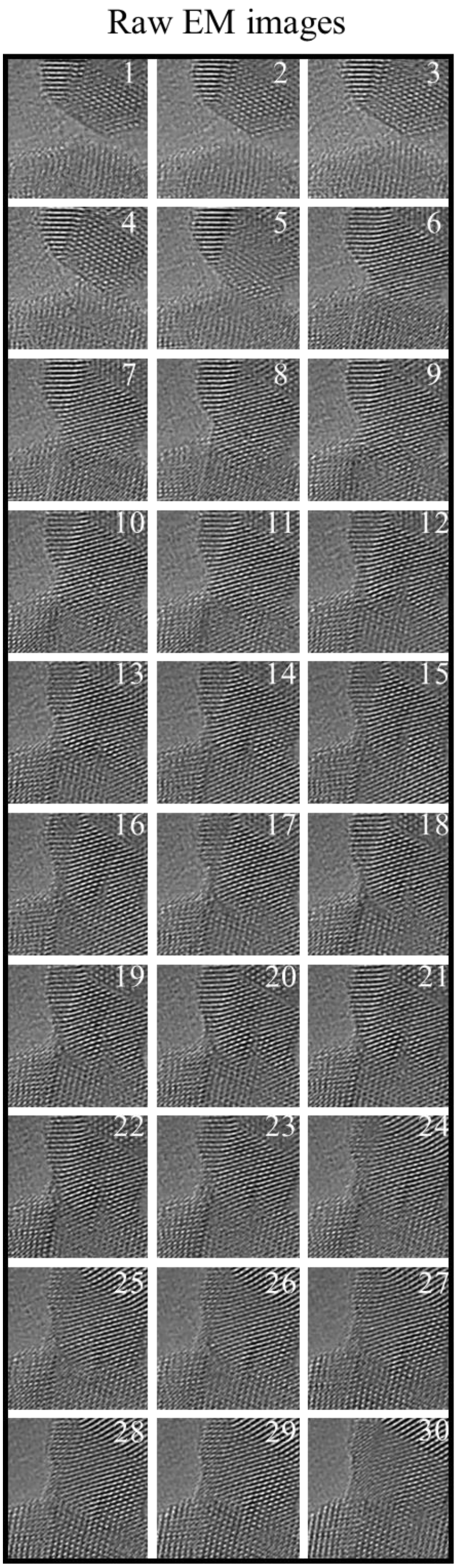

Figure S6. Raw images of Figure 2.
